# SRSF1 and PTBP1 Are *trans*-Acting Factors That Suppress the Formation of a CD33 Splicing Isoform Linked to Alzheimer’s Disease Risk

**DOI:** 10.1128/MCB.00568-18

**Published:** 2019-08-27

**Authors:** Petra van Bergeijk, Uthpala Seneviratne, Estel Aparicio-Prat, Robert Stanton, Samuel A. Hasson

**Affiliations:** a Internal Medicine Research Unit, Pfizer, Cambridge, Massachusetts, USA; b Computational Sciences, Pfizer, Cambridge, Massachusetts, USA; c Chemical Biology and Medicine Design, Pfizer, Cambridge, Massachusetts, USA

**Keywords:** Alzheimer’s disease, CD33, antisense oligonucleotides, functional genomic screen, siRNA, splicing

## Abstract

A single nucleotide polymorphism (SNP) in exon 2 of the CD33 gene is associated with reduced susceptibility to late-onset Alzheimer’s disease (AD) and causal for elevated mRNA lacking exon 2. In contrast to full-length CD33, transcripts lacking exon 2 result in CD33 protein unable to suppress activation responses in myeloid cells, including microglia. Currently, little is known about the regulation of CD33 exon 2 splicing. Using functional genomics and proteomic approaches, we found that SRSF1 and PTBP1 act as splicing enhancers to increase CD33 exon 2 inclusion in mRNA. Binding of PTBP1 to RNA sequences proximal to the intron 1-exon 2 splice junction is altered by the SNP and represents a potential mechanism behind the SNP-genotype dependent alternative splicing. Our studies also reveal that binding of SRSF1 to the CD33 RNA is not altered by the SNP genotype. Instead, a putative SRSF1 binding sequence at the 3′ end of exon 2 directs CD33 exon 2 inclusion into the mRNA, indicating that PTBP1 and SRSF1 promote full-length isoform expression through different mechanisms. Our findings shed light on molecular interactions that regulate CD33 exon 2 splicing, ultimately impacting receptor expression on the cell surface. These data aid in the understanding of CD33’s regulation of microglial signaling underpinning the AD genetic associations.

## Abstract (precis)

A single nucleotide polymorphism (SNP) in exon 2 of the CD33 gene is associated with reduced susceptibility to late-onset Alzheimer’s disease (AD) and causal for elevated mRNA lacking exon 2. In contrast to full-length CD33, transcripts lacking exon 2 result in CD33 protein unable to suppress activation responses in myeloid cells, including microglia. Currently, little is known about the regulation of CD33 exon 2 splicing.

## INTRODUCTION

Alzheimer’s disease (AD) is a progressive neurodegenerative disease characterized by the deposition of amyloid beta plaques and neurofibrillar tangles, as well as microglia-mediated neuroinflammation. Microglia are the resident myeloid cells of the central nervous system and play a key role in innate immune functions in the brain (1). Recent genome-wide association studies (GWAS) implicate receptors expressed selectively on microglia as significant modifiers of AD risk, presumably through the regulation of neuroimmune responses (2–6).

Among single nucleotide polymorphisms (SNPs) identified as modifiers of late-onset AD risk, rs3865444 is associated with the CD33 gene, endowing carriers with a modestly protective odds ratio (5–9). The initially identified AD GWAS rs3865444^A^ SNP is located in the CD33 promoter (5–7, 9) and vigorously correlated with the increase of a common isoform lacking exon 2 (referred to as the exon 2 “skipped” isoform) in blood (10). Follow-up studies employing minigenes demonstrated that differential CD33 splicing associated with rs3865444 genotype (C→A) could be functionally attributed to a strongly coinherited (linkage disequilibrium = 1) rs12459419 SNP (C→T), located at the fourth base of exon 2 (11, 12).

The CD33 gene encodes a transmembrane receptor that binds to sialic acids, which are present on extracellular glycoproteins and glycolipids (13). Since CD33 is expressed throughout the myeloid lineage, it has been targeted as a molecular beacon in large-molecule therapeutics directed against acute myeloid leukemia. In the brain, CD33 is selectively expressed by microglia, where it can suppress microglial activation phenotypes such as cytokine secretion and phagocytosis in response to AD-relevant stimuli (14, 15). The extracellular IgV domain of CD33 is encoded entirely by exon 2 and mediates sialic acid binding (reviewed in references 16 and 17). CD33 protein resulting from mRNA isoforms lacking exon 2 were functionally unable to suppress microglia activation and amyloid plaque phagocytosis *in vitro* and *in vivo* (18, 19). CD33 levels were also shown to be elevated in postmortem AD brain samples compared to nondemented controls (18), suggesting that functional CD33 is correlated with AD risk and pathogenesis.

Since full-length CD33 expression may inhibit the signaling of microglial receptors such as TREM2, pharmacologically altering exon 2 splicing is a potential therapeutic avenue for AD. However, it is unknown how the alternative splicing of exon 2 is regulated. In general, control of alternative splicing events is mediated by *trans*-acting splicing regulators that bind to *cis*-regulatory sequences within or near the regulated exon. Splicing factors that bind to the pre-mRNA act bidirectionally to enhance or silence splicing based on (i) the properties of the protein, (ii) the binding location relative to the alternatively spliced exon, and (iii) whether it cooperates or competes with other splicing enhancers or silencers that may function in the same region (20).

Here, we aimed to identify the *trans*-acting splicing regulators, as well as the *cis*-regulatory sequences involved in alternative CD33 exon 2 splicing. We report that serine and arginine rich splicing factor 1 (SRSF1) and polypyrimidine tract binding protein 1 (PTBP1) are enhancers of CD33 exon 2 inclusion in expressed mRNA isoforms. PTBP1 binding to the CD33 RNA intron 1-exon 2 splice junction is reduced in the presence of the AD-associated rs12459419^T^ SNP. Given that PTBP1, in addition to its role as a splicing repression, has been shown to enhance the inclusion of many alternatively spliced exons, loss of its binding at the 5′ of exon 2 would explain the observed SNP-associated elevation of exon 2 skipping. Furthermore, a combination of siRNA screening against known splicing factors and RNA scanning with methoxyethyl (MOE) modified antisense oligonucleotides led to the identification of SRSF1 as another *trans*-acting factor important for CD33 exon 2 splicing. In contrast to PTBP1, the rs12459419 AD-associated SNP did not affect the binding of SRSF1 to the CD33 RNA intron 1-exon 2 splice junction. Instead, disrupting or partially blocking an *in silico* predicted SRSF1 binding site at the 3′ end of exon 2 increased exon 2 skipped mRNA transcripts. Thus, we show that SRSF1 and PTBP1 can act to enhance full-length CD33 transcript expression and that modulating their specific interactions with CD33 pre-mRNA can alter protein levels on the cell surface.

## RESULTS

### The rs12459419 SNP affects CD33 mRNA and protein levels exclusively through alternative splicing.

**FIG 1:**
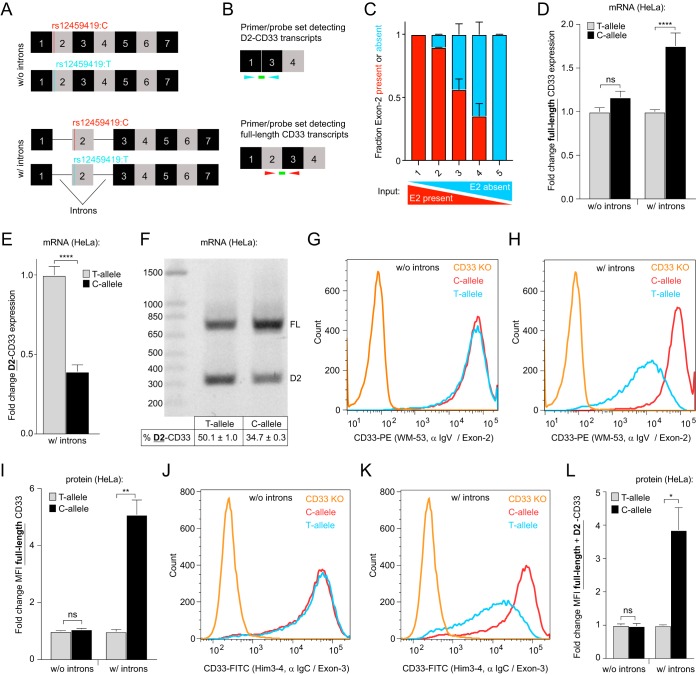
The rs12459419 SNP genotype does not affect CD33 mRNA or protein levels in CD33 cDNAs lacking introns. (A) Overview of the CD33 expression constructs used. (B) Overview of the RT-qPCR primer and probe sets used to detect the various CD33 mRNA transcripts. (C) Validation of the RT-qPCR assays ability to quantify CD33 exon 2 splicing using gBlocks that represent D2-CD33 or full-length CD33 cDNA. The fraction of detected exon 2 included (red) or exon 2 skipped (cyan) gene fragments is shown. Input quantities of the two different CD33 fragments are indicated below the graph. (D and E) mRNA levels of exon 2 included (D) and exon 2 skipped (E) CD33 transcripts normalized to the corresponding T-allele in HeLa cells transfected with the CD33 constructs shown in panel A, measured by RT-qPCR. (F) The RT-PCR products from a single primer set that simultaneously detects full-length CD33 (FL) and D2-CD33 in HeLa cells transfected with the CD33 constructs, including introns (A), visualized on a 1.2% agarose gel. The percent D2-CD33 values ± the standard errors of the mean (SEM) are indicated below each lane. (G to L) Flow cytometry analysis of CD33 surface levels using FITC- or PE-labeled antibodies targeting exon 2 (WM-53 [G and H]) or exon 3 (Him3-4 [J and K]) of CD33 in HeLa cells transfected with the CD33 constructs shown in panel A. CD33 KO THP1 cells were utilized as a negative control. α IgV, anti-IgV; α IgC, anti-IgG C_2_ domain. The mean fluorescence intensity (MFI) normalized to the corresponding T-allele is depicted in panels I and L. Error bars indicate means + the SEM. A two-tailed *t* test was performed. *, *P* ≤ 0.05; **, *P* ≤ 0.01; ****, *P* ≤ 0.0001; ns, not significant. *n* = 3 biological replicates for each plot.

Recent work has established that the AD-associated DNA polymorphism rs12459419 in CD33 is responsible for altered exon 2 splicing (11). The neutral rs12459419^C^ allele showed larger amounts of full-length CD33 compared to the protective rs12459419^T^ allele. To confirm earlier reports (11) and rule out that the SNP may alter full-length CD33 mRNA and/or protein levels via other mechanisms, we created CD33 expression constructs containing the rs12459419 polymorphism in the presence or absence of introns (Fig. 1A). We hypothesized that full-length CD33 cDNAs lacking introns would display SNP-dependent alterations of mRNA and/or protein levels if a nonsplicing mechanism such as nuclear export, mRNA stability, or translation was driving the phenotype.

To test this, HeLa cells were transfected with the CD33 expression constructs shown in Fig. 1A and reverse transcription-quantitative PCR (RT-qPCR) assays (Fig. 1B) were utilized to quantify changes in the levels of full-length and exon 2 absent (D2-CD33) CD33 mRNA. Our RT-qPCR assays were validated using titration of synthetic DNA fragments (gBlocks) that contained full-length or D2-CD33 coding sequences (Fig. 1C). We observed that when cells were transfected with CD33 constructs lacking introns, the rs12459419 genotype had no significant effect on CD33 expression (Fig. 1D). In the presence of introns, the expression of full-length CD33 mRNA was a significant 1.8-fold higher in rs12459419^C^ compared to rs12459419^T^ construct-transfected cells (Fig. 1D), and the D2-CD33 isoform expression correspondingly decreased in a rs12459419 genotype-dependent manner (Fig. 1E). To align our HeLa cell-based CD33 splicing assays with previous RT-PCR characterizations of CD33 minigenes carrying rs12459419 SNPs, we examined the CD33 splicing via an RT-PCR assay using a single primer set that simultaneously detected full-length and D2-CD33 (Fig. 1F). After preparing cDNA from HeLa cells transfected with intron-containing rs12459419^C^ or rs12459419^T^ CD33 expression constructs, gel electrophoresis of the PCR products demonstrated the characteristic elevation of CD33-D2 in the presence of rs12459419^T^ (Fig. 1F). The HeLa cell system therefore recapitulated previous rs12459419 genotype-dependent minigene splicing in myeloid cells (11).

Since CD33 is normally trafficked to the cell surface as a single-pass transmembrane receptor, flow cytometry analysis using a labeled anti-CD33 antibody recognizing the IgV domain (WM-53) allowed us to examine whether CD33 surface levels reflect mRNA expression. In accordance with our RT-qPCR mRNA expression observations, without introns, rs12459419 genotype had no impact on cell surface CD33 protein levels (Fig. 1G and I). However, our rs12459419^C^ construct with introns resulted in 5.1-fold higher cell surface IgV domain-containing CD33 protein levels than rs12459419^T^ construct-transfected cells (Fig. 1H and I). Interestingly, similar increases of total CD33 protein at the cell surface were obtained when we repeated the flow assay using an anti-CD33 antibody specific for the extracellular C_2_ IgG domain encoded by exon 3 (Him3-4 [21]) (Fig. 1J to L). Given that the C_2_ domain is present in both CD33 splice variants, this suggests that D2-CD33 proteins are less represented in the population of cell surface CD33 compared to full-length isoforms. Taken together, our RT-qPCR and flow cytometry assays indicate that the rs12459419 genotype impact on CD33 expression requires introns and is driven through a splicing-mediated mechanism.

### SRSF1 directs CD33 exon 2 inclusion into spliced mRNAs.

**FIG 2:**
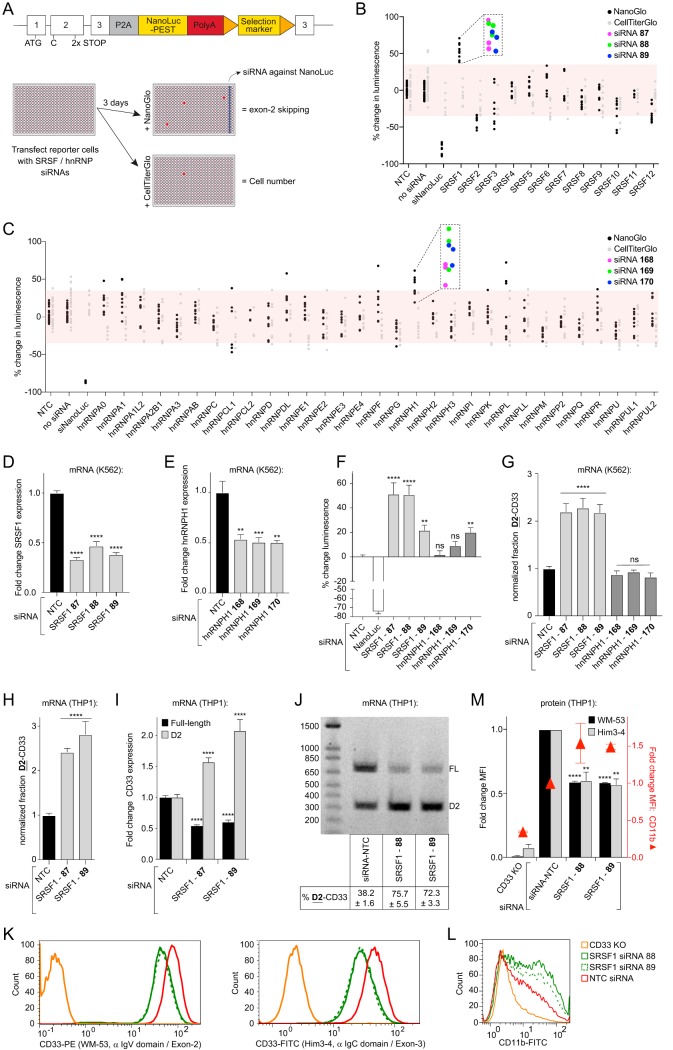
SRSF1 knockdown increases CD33 exon 2 skipping. (A) Overview of the splicing reporter incorporated at the endogenous CD33 locus in K562 cells (top), as well as the siRNA screening strategy to identify modifiers of CD33 exon 2 splicing (bottom). (B and C) Percent change in luminescence upon knockdown of the SRSF family members (B) or hnRNP family members (C) in the splicing reporter cell line compared to NTC-transfected cells. For each gene, three different siRNAs were tested in triplicate, except for SRSF11, hnRNPCL1, and hnRNPH2, where two different siRNAs were tested, and hnRNPCL2, where a single siRNA was tested, due to the availability of predesigned siRNAs. Red boxes mark ±35% compared to the NTC. Each dot represents a single well. Purple, green, and blue dots correspond to unique siRNA reagents. (D and E) Relative mRNA levels of SRSF1 transcripts (D) or hnRNPH1 transcripts (E) in K562 cells upon knockdown of the corresponding genes, measured by RT-qPCR compared to the NTC. (F) Percent change in luminescence upon knockdown of SRSF1 and hnRNPH1 in the splicing reporter cell line compared to NTC-transfected cells. (G and H) The fraction of total CD33 transcripts lacking exon 2 in K562 cells (G) and THP1 monocytes (H) upon knockdown of SRSF1 and/or hnRNPH1 compared to the NTC, as measured by RT-qPCR. (I) Fold change of full-length CD33 and D2-CD33 mRNA transcripts in THP1 monocytes upon knockdown of SRSF1 compared to the NTC. (J) RT-PCR products from a single primer set that simultaneously detects full-length CD33 (FL) and D2-CD33 (D2) in THP1 monocytes transfected with the NTC or upon knockdown of SRSF1, visualized on a 1.2% agarose gel. The percent D2-CD33 values ± the SEM are indicated below each lane. (K to M) Flow cytometry analysis of CD33 and CD11b surface levels using FITC- or PE-labeled antibodies targeting CD33 exon 2 (K, left) or exon 3 (K, right) or CD11b (L) in THP1 monocytes upon knockdown of SRSF1. CD33 KO cells were utilized as a negative control. The NTC normalized mean fluorescence intensity (MFI) is quantified in M. A one-way ANOVA was performed. **, *P* ≤ 0.01; ***, *P* ≤ 0.001; ****, *P* ≤ 0.0001; ns, not significant. NTC, nontargeting control. Error bars indicate means plus the SEM. *n* = 3 biological replicates for each plot.

Alternative splicing is a highly regulated process by the activities of *trans*-acting splicing regulators. These splicing regulators include the serine- and arginine-rich splicing factors (SRSFs) and the heterogeneous nuclear ribonucleoproteins (hnRNPs) that bind to *cis*-regulatory sequences in the pre-mRNA to modify splicing (20). To identify splicing factors that modify exon 2 splicing, we systematically knocked down the expression of known SRSFs and hnRNPs in a cell-based small interfering RNA (siRNA) screen. The screen was performed on a K562 myeloid cell line that endogenously expresses CD33 and contains a CD33 exon 2 splicing reporter. In these cells, stop codons were genome edited into exon 2 of the genomic CD33 locus, along with a NanoLuc luciferase reporter gene inserted in frame with the third exon. Tandem stop codons were included to minimize aberrant readthrough that may occur. The endogenous splicing reporter cell line was designed as a bicistronic (2A) system so that NanoLuc-mediated luminescence quantitatively tracks the amount of CD33 mRNA lacking exon 2 (Fig. 2A, top). In the screen, cells were reverse transfected with siRNAs (see the supplemental material) arrayed in 384-well plates and NanoLuc signal expression was measured 72 h posttransfection by adding a NanoLuc enzyme substrate (see Materials and Methods). As siRNA knockdown of splicing factor genes could result in cell toxicity, we monitored changes in cell viability using a parallel luminescent cell viability assay in replicate plates (Fig. 2A, bottom). With an emphasis on testing multiple unique siRNA reagents for each target, we screened knockdowns of SRSF1-12 and 31 different hnRNPs. Each 384-well plate included an array of nontargeting control (NTC) siRNAs as well. A given siRNA was called “active” by the following criteria: (i) the average NanoLuc signal from three technical replicates differed by more than 35% from the NTC, (ii) the strictly standardized mean difference (SSMD) was <−1.5 or >1.5 (22), and (iii) the 35% threshold was still met after correcting for changes in cell viability. At the gene level, candidate hits were selected from targets with multiple unique siRNA reagents that met the active threshold. Our screen identified the knockdown of SRSF1 and hnRNPH1 as promising candidates (three of three unique siRNA reagents tested for each gene met active criteria) (Fig. 2B and C), and these genes were selected for further analysis (Fig. 2D and E). Whereas retesting revealed a significant increase in luminescence from the CD33 splicing reporter using each of the three SRSF1 siRNAs, only one of three siRNAs targeting hnRNPH1 induced a significant increase in luminescence (Fig. 2F).

To orthogonally validate the siRNA screening results, we developed RT-qPCR assays capable of distinguishing splice isoforms and translate observed increases in reporter luminescence into elevated levels of exon 2 skipped transcript (Fig. 1B and C). We noted that in the CD33 splicing reporter cells, the basal fraction D2-CD33 was ∼20% higher in transcripts derived from the genome-edited CD33 locus compared to transcripts derived from the wild-type allele (data not shown). This indicates that the engineered allele may alter some of the *cis* elements contributing to CD33 exon 2 splicing patterns or that the stop codons used to gate the luciferase reporter translation in full-length CD33 transcripts sensitize the mRNA for degradation, for example, via nonsense-mediated mRNA decay. To eliminate potential artifacts from the splicing reporter, confirmatory knockdowns and RT-qPCR assays were performed in unedited K562 cells that endogenously express CD33 (rs12459419^T/T^ genotype). SRSF1 but not hnRNPH1 knockdown significantly increased the fraction of total CD33 transcript expressed as D2-CD33 (Fig. 2G). Because CD33 in the brain is specifically expressed on microglia derived from the myeloid lineage (11, 23), we also tested the effect of SRSF1 knockdown on CD33 splicing in myelogenic THP1 monocytes as a second cellular model (rs12459419^C/C^). The fraction of total CD33 mRNA message expressed as D2-CD33 increased significantly 72 h post-transfection with both siRNAs 87 (2.4-fold) and 89 (2.8-fold) (Fig. 2H).

**FIG 3:**
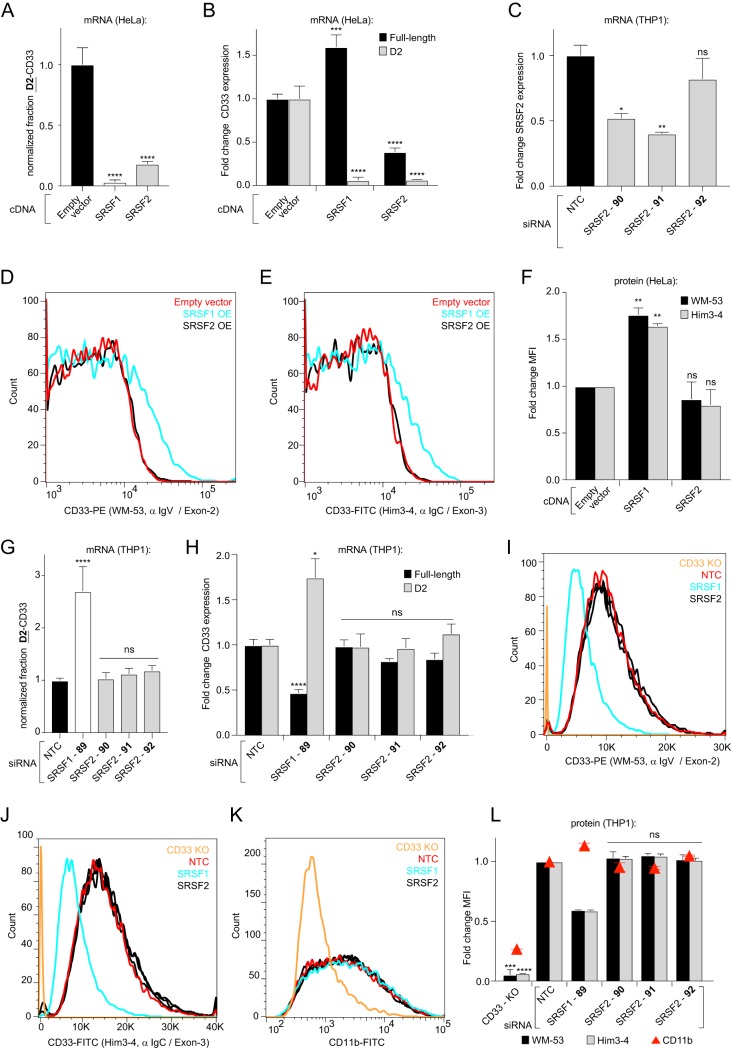
SRSF1 but not SRSF2 levels drive the amounts of full-length and D2-CD33 mRNA transcripts in opposite directions and modulate the quantity of CD33 protein at the cell surface. (A and B) Fraction of CD33 mRNA transcripts expressed as D2-CD33 in HeLa cells overexpressing SRSF1 or SRSF2, normalized to the empty vector control (A). In the same samples, mRNA levels of CD33 exon 2 included (black bars) and exon 2 skipped (gray bars) mRNA transcripts were measured by RT-qPCR (B). (C) Relative mRNA levels of SRSF2 transcripts in THP1 monocytes upon knockdown, measured by RT-qPCR compared to the NTC. (D to F) Flow cytometry analysis of CD33 protein surface levels using labeled antibodies targeting CD33 exon 2 (D) or exon 3 (E) in HeLa cells coexpressing SRSF1 or SRSF2 with a CD33 minigene. The mean fluorescence intensities (MFI) are quantified in panel F and were normalized to control cells expressing the CD33 minigene only. (G and H) Fraction of CD33 mRNA transcripts expressed as D2-CD33 upon SRSF1 or SRSF2 knockdown in THP1 monocytes, normalized to cells transfected with an NTC siRNA (G). In the same samples, NTC-normalized levels of full-length CD33 and D2-CD33 were determined by RT-qPCR (H). (I to L) Flow cytometry analysis of CD33 and CD11b protein surface levels using FITC- or PE-labeled antibodies targeting CD33 exon 2 (I) or exon 3 (J) or CD11b (K) in THP1 monocytes upon knockdown of SRSF1 or SRSF2. CD33 KO cells were utilized as a negative control. The NTC-normalized MFIs are shown in panel L. Error bars indicate means plus the SEM (A, B, F, G, H, and L) or medians + the SEM (C). A one-way ANOVA was performed for each plot. *, *P* ≤ 0.05; **, *P* ≤ 0.01; ***, *P ≤* 0.001; ****, *P* ≤ 0.0001; ns, not significant. *n* = 3 biological replicates for each plot. α IgV, anti-IgV; α IgC, anti-IgC_2_ domain.

We hypothesized that the mechanism behind the observed D2-CD33 message increase could be due to SRSF1 knockdown causing either (i) an induction of total CD33 message or (ii) a change in the ratio of expressed D2-CD33-to-full-length CD33. To test this hypothesis, we independently measured the fold change in D2-CD33 and full-length CD33 mRNA levels after SRSF1 siRNA transfection compared to the NTC-transfected cells. This analysis revealed 1.6- and 2.1-fold increases in the D2-CD33 levels for siRNAs 87 and 89, respectively, compared to NTC siRNA (Fig. 2I). Full-length CD33 mRNA levels were 1.9- and 1.7-fold lower in the same samples (Fig. 2I), indicating that the fraction of full-length transcripts was decreasing concomitantly with the increase in D2-CD33 mRNA. Visualizing the amplicons of a single RT-PCR that simultaneously detected full-length and D2-CD33 on an agarose gel corroborated the RT-qPCR data and showed similar changes in CD33 exon 2 splicing upon SRSF1 knockdown (Fig. 2J). CD33 protein levels at the cell surface, as measured by flow cytometry, reflected mRNA results, as we observed a decrease upon SRSF1 knockdown for both full-length (Fig. 2K [left] and 2M) and total CD33 levels (Fig. 2K [right] and 2M). To ensure that lower CD33 levels are neither due to cell toxicity nor due to a general decrease in the cell surface protein level, we included the detection of the macrophage/microglial marker CD11b. Upon SRSF1 knockdown, we observed depletion of cell surface CD33 protein but not CD11b (Fig. 2L and M). This indicates that the observed decrease in CD33 surface levels is not due to a general decrease in cell surface protein levels or cell toxicity but rather is a selective consequence of SRSF1 knockdown. Reciprocally, the overexpression of SRSF1 cDNA in HeLa cells resulted in a 1.6-fold-higher expression of full-length CD33 mRNA, whereas the levels of D2-CD33 were 16.3-fold lower relative to empty vector-transfected cells (Fig. 3A and B). Our subsequent flow cytometry assay of cells overexpressing SRSF1 demonstrated similar mean increases in total (1.6-fold) and full-length CD33 (1.8-fold) protein levels at the cell surface compared to empty-vector-transfected cells (Fig. 3D to F). Thus, we observed that the SRSF1 splicing factor promotes CD33 exon 2 inclusion in cultured K562 cells and THP1 monocytes.

Previous *in silico* analysis has predicted that rs12459419^T^ alters a putative SRSF2 binding site in the CD33 pre-mRNA (11). However, SRSF2 knockdown did not cross the selection threshold in our siRNA screening with the luminescent splicing reporter cells (Fig. 2B). To further test the potential participation of SRSF2 in CD33 exon 2 splicing, we subjected the SRSF2 siRNA knockdown in unmodified THP1 monocytes to the RT-qPCR and flow cytometry analyses. We tested three unique SRSF2 siRNAs that diminished SRSF2 mRNA transcripts by 48, 60, and 18%, respectively (Fig. 3C). In contrast to SRSF1 silencing, the knockdown of SRSF2 did not alter the relative amounts of the CD33 isoforms at the mRNA level (Fig. 3G and H). SRSF2 knockdown also did not alter CD33 V-domain containing protein levels at the cell surface (Fig. 3I and L), total cell surface CD33 protein (Fig. 3J and L), and CD11b levels (Fig. 3K and L). Unexpectedly, SRSF2 cDNA overexpression in HeLa cells significantly reduced the fraction of total CD33 transcript expressed as D2-CD33 (Fig. 3A). However, SRSF2 overexpression also depleted the total CD33 mRNA levels, as we observed both exon 2 skipped (15.2-fold reduced) and exon 2 included (2.6-fold reduced) transcripts being diminished (Fig. 3B) compared to cells transfected with an empty vector. Even though SRSF2 cDNA overexpression depleted all CD33 mRNA transcripts in our HeLa cell splicing assay, this reduction did not result in a significant change in full-length CD33 protein level at the plasma membrane compared to control cells transfected with an empty vector (Fig. 3D to F). In addition to lowering CD33 mRNA levels, SRSF2 might increase CD33 translation, protein stability, or CD33 trafficking to the plasma membrane, resulting in unaltered CD33 protein surface levels. Although future experiments would be necessary to determine the mechanism by which SRSF2 affects CD33 mRNA and/or protein levels, our results suggest that SRSF2 may be involved in overall CD33 splicing and yet is not a selective factor for exon 2 splice site recognition and isoform determination. Taken together, our siRNA screening and follow-up experiments demonstrate that SRSF1 but not SRSF2 stimulates exon 2 inclusion into the CD33 mRNA.

### A *cis*-regulatory exon 2 splicing enhancer sequence that overlaps with a putative SRSF1 binding site is located near the 3′ end of CD33 exon 2.

**FIG 4:**
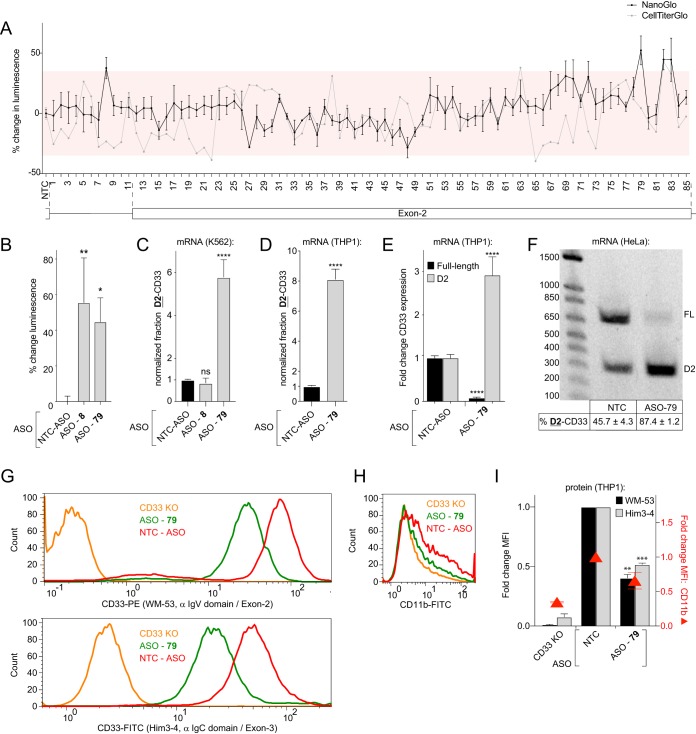
Treatment with a 15-nucleotide MOE-ASO that targets near the 3′ end of exon 2 increases CD33 exon 2 skipping. (A) Percent change in luminescence upon transfection of K562 splicing reporter cells with MOE-ASOs targeting the CD33 RNA. Red boxes mark ±35% compared to the NTC. (B) Percent change in luminescence from K562 splice reporter cells transfected with MOE-ASOs 8 and 79 compared to the NTC. (C and D) Fraction of CD33 mRNA transcripts lacking exon 2, based on RT-qPCR analysis in K562 cells (C) and THP1 monocytes (D) upon MOE-ASO treatment, compared to the NTC. (E) NTC-normalized expression of CD33 exon 2 present (black bars) and exon 2 absent (gray bars) mRNA transcripts in THP1 monocytes upon treatment with ASO 79. (F) RT-PCR products from a single primer set that simultaneously detects full-length CD33 (FL) and D2-CD33 (D2) in THP1 monocytes transfected with the NTC or MOE-ASO 79, visualized on a 1.2% agarose gel. The percent D2-CD33 values ± the SEM are indicated below each lane. (G to I) Flow cytometry analysis of CD33 and CD11b surface levels using FITC- or PE-labeled antibodies targeting CD33 exon 2 (G, top) or exon 3 (G, bottom) or CD11b (H) in THP1 monocytes upon knockdown of SRSF1 or treatment with MOE-ASO 79. CD33 KO cells were utilized as a negative control. α IgV domain, anti-IgV domain; α IgC domain, anti-IgC_2_ domain. The NTC-normalized MFIs are quantified in panel I. A one-way ANOVA (B, C, and I) and a two-tailed *t* test (D and E) were performed. *, *P* ≤ 0.05; **, *P* ≤ 0.01; ***, *P* ≤ 0.001; ****, *P* ≤ 0.0001. NTC, nontargeting control. Error bars indicate means plus the SEM. *n* = 3 biological replicates for each plot.

To identify *cis*-regulatory splicing enhancer and silencer sequences within the CD33 transcript that regulate exon 2 splicing, we tiled 2′-methoxyethyl (MOE) antisense oligonucleotides (ASOs) along exon 2 and the upstream intron. This strategy has been used previously to identify functional binding sites of proteins with RNA (24). The ASOs anneal to the RNA and form a site-specific steric hindrance to protein binding, but do not induce RNA degradation because the MOE modification blocks the recognition by RISC and RNase H (25). We hypothesized that MOE-ASO-mediated blocking of splicing regulator binding to sequences within the pre-mRNA could reveal *cis*-regulatory elements involved in exon 2 splicing. Previous reports have identified MOE-ASOs that modify the splicing of exon 7 in SMN2 mRNA transcripts by blocking the action of *cis*-regulatory elements (24). Using these SMN2-directed MOE-ASOs as positive controls, we optimized our in-house methodology to deliver this type of oligonucleotide into K562 cells and effectively alter mRNA splicing. As SMN2 is expressed in K562 cells, we found that transfecting known MOE-ASOs that were designed to modulate SMN2 exon 7 splicing (24) resulted in >20% increases in exon 7 inclusion (data not shown). Next, we transfected an array of MOE-modified ASO reagents into the K562 CD33-splicing reporter cell line in a similar screen as with the siRNAs (Fig. 4A). Our tiling array of MOE-modified ASOs covered the CD33 exon 2 region with a maximum increment of 8 bp. MOE-ASOs 49 to 51 cover the introduced stop codons in exon 2 of the splicing reporter and therefore might bind with lower affinity to the CD33 reporter locus compared to the unedited allele. The screen yielded three regions in the RNA that, when blocked with an MOE-ASO, increased reporter luminescence over 35% with an SSMD >1.5 or <–1.5 (Fig. 4A). Our parallel cell viability assay indicated that the change in reporter luminescence for the active reagents could not be explained by an increase in viable cells for MOE-ASOs 8 and 79 (Fig. 4A). We subsequently reconfirmed a significant increase in splicing reporter luminescence for cells transfected with ASOs 8 and 79 (Fig. 4B). Returning to the parental K562 cell line lacking the genome-edited reporter, RT-qPCR analysis demonstrated that following MOE-ASO 79 transfection, the fraction of total CD33 mRNA expressed as D2-CD33 was elevated 5.8-fold compared to nontargeting control MOE-ASO (Fig. 4C). Transfection of THP1 monocytes with MOE-ASO 79, which targets the end of exon 2 (AAGTACCAAATACAG), yielded similar results (Fig. 4D). We next determined the D2-CD33 and full-length CD33 mRNA levels compared to the NTC using RT-qPCR to determine the cause of the altered isoform fraction. In MOE-ASO 79-transfected cells, D2-CD33 levels were 2.9-fold higher, whereas full-length CD33 levels were 13.3-fold lower compared to cells transfected with the control MOE-ASO (Fig. 4E). These results were further validated by the agarose gels that visualized the amplicons from RT-PCR spanning exons 1 to 3. Gel electrophoresis showed a decrease in full-length transcript concomitant with an increase in D2-CD33 in MOE-ASO 79-transfected cells (Fig. 4F). Flow cytometry of MOE-ASO 79-transfected THP1 cells demonstrated that CD33 cell membrane protein levels mirrored changes in mRNA levels and were significantly decreased 2.0-fold (Fig. 4G to I). Although MOE-ASO 79-transfected THP1 cells show slightly decreased CD11b levels compared to the NTC, this decrease was not significant and is minor compared to changes observed in CD33 levels. This indicates that MOE-ASO 79-transfection selectively reduces cell surface CD33 protein levels (Fig. 4G to I). Thus, blocking the AAGTACCAAATACAG sequence in CD33 exon 2 using an MOE-ASO increases exon 2 skipping in the CD33 mRNA, indicating this sequence is part of an exonic splicing enhancer.

**FIG 5:**
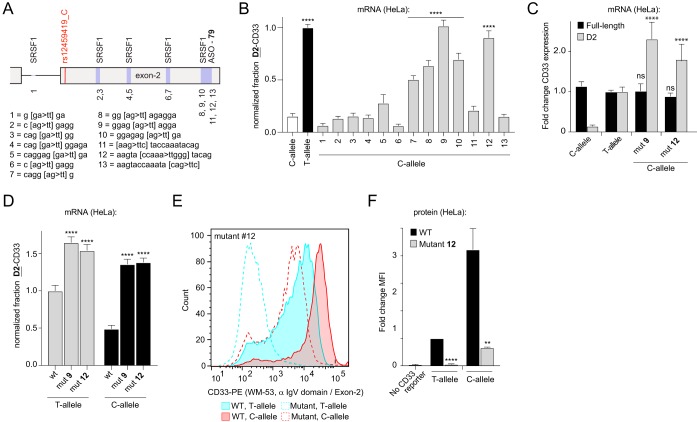
Disrupting a predicted SRSF1 binding site near the 3′ end of exon 2 increases CD33 exon 2 skipping. (A) Overview of the predicted SRSF1 binding sites, as well as the ASO targeting sequence in CD33 exon 2. The numbered key indicates the identity of binding/targeting site mutants used in panel B. (B and D) T-allele-normalized fraction of CD33 mRNA transcripts lacking exon 2, as measured by RT-qPCR in HeLa cells transfected with CD33 minigenes carrying mutations as shown in panel A. (C) Fold change of full-length CD33 and D2-CD33 mRNA transcripts in HeLa cells overexpressing a mutant CD33 minigene compared to WT CD33 minigene-overexpressing cells, as measured by RT-qPCR. (E and F) Flow cytometry analysis of CD33 surface levels using a labeled antibody targeting CD33 C_2_ domain in HeLa cells overexpressing WT or mutant 12 containing CD33 minigenes in combination with either rs12459419^C^ or rs12459419^T^. The MFIs were normalized to cells transfected with WT-CD33 minigenes carrying rs12459419^T^. Error bars indicate means plus the SEM. A one-way ANOVA (B to D) and a two-tailed *t* test (F) were performed. **, *P* ≤ 0.01; ****, *P* ≤ 0.0001; ns, not significant. *n* = 3 biological replicates for each plot.

To determine whether our finding that SRSF1 functionally modulated CD33 exon 2 splicing intersected with results from the MOE-ASO scanning study, we searched for predicted SRSF1 binding sites in exon 2 and the upstream intron. SRSF1 has been shown to bind purine-rich sequences (26–33), and sequence analysis yielded five putative SRSF1 binding sites. To determine whether any of these predicted SRSF1 binding sites in the exon 2 pre-mRNA region were functional, HeLa cells were sequentially transfected with a set of intron-containing CD33 expression minigenes that carried the rs12459419^C^ allele, along with mutations in each candidate consensus sequence (Fig. 5A). Given that the rs12459419 SNP-dependent exon 2 splicing phenotype observed in myeloid cells can be recapitulated in HeLa cells exogenously expressing intron-containing CD33 minigenes (Fig. 1D to F), we utilized HeLa cells as a platform to rapidly probe the impact of *cis*-acting elements and *trans*-acting factors. In accordance with our MOE-ASO screening data (Fig. 4A), disrupting putative SRSF1 binding sites proximal to the AD-associated SNP at the start of exon 2 did not significantly alter the level of exon 2 skipping (Fig. 5B). In contrast, mutations in both the MOE-ASO 79-targeted site and the predicted SRSF1 recognition sequencing neighboring/overlapping with the ASO-targeted site, located at the end of exon 2, significantly increased CD33 mRNA exon 2 skipping (Fig. 5B). We retested mutant 9 and 12 constructs that in the minigene screen had shown the highest increase in CD33 exon 2 skipping and confirmed these results (data not shown). Furthermore, compared to the WT CD33 expression construct, mutant 9 and 12 constructs increased the amounts of D2-CD33 by 16.1- and 12.6-fold, respectively (Fig. 5C). These results suggest that CD33 exon 2 splicing is regulated by *cis* elements at the 3′ end of exon 2 that recruit SRSF1 to enhance exon 2 inclusion in the spliced transcripts.

Because the identified splicing enhancer sequence is separated by more than 300 bp from the AD-associated SNP, we wondered whether its functional effect on alternative exon 2 splicing is dependent on rs12459419 genotype. Therefore, we created new intron-containing CD33 expression constructs that combined the predicted SRSF1 recognition site mutants 9 and 12 with either rs12459419^T^ or rs12459419^C^ SNP variants. When HeLa cells were transfected with rs12459419^T^-containing minigenes, both exon 2 3′ mutations further increased the fraction of CD33 mRNA expressed as D2-CD33 by a significant 1.4-fold compared to the wild-type rs12459419^T^ minigene. However, the rs12459419 genotype-dependent effects on exon 2 splicing largely disappeared in minigenes containing either mutant 9 or 12 (Fig. 5D). CD33 protein levels at the cell surface reflected mRNA results as we observed that mutation 12 decreased full-length CD33 levels in both rs12459419^T^ (18-fold) and rs12459419^C^ (6.4-fold) minigene transfected HeLa cells (Fig. 5E and F). Proteins derived from minigenes carrying mutation 9 could not be detected by WM-53 since the known V-domain epitope (21) is likely disrupted by the mutation. Together, these data indicate that disrupting the predicted SRSF1 binding site or the ASO targeting site at the end of exon 2 increases skipping independent of the AD-associated SNP.

### The binding of PTBP1 to the intron-exon junction at the 5′ of exon 2 is reduced in RNA fragments carrying the rs12459419^T^ allele.

**FIG 6:**
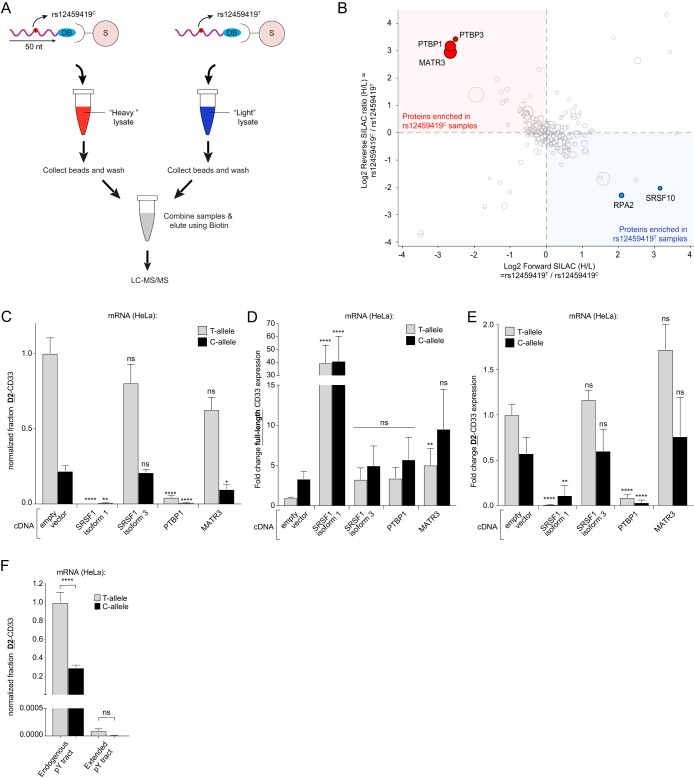
PTBP1 shows a reduced binding to the intron-exon junction at the 5′ of exon 2 in RNA fragments carrying the rs12459419^T^ allele. (A) Workflow for the reverse SILAC LC-MS/MS analysis of the CD33 RNA fragment pulldown. DB, desthiobiotin; S, streptavidin. (B) Log_2_ ratio of the reverse (*y* axis) and forward (*x* axis) SILAC experiments. Red dots indicate proteins that are >4-fold enriched in the rs12459419^C^ pulldown, whereas blue dots indicate proteins that are >4-fold more abundant in the rs12459419^T^ samples. The sizes of the circles correspond to the number of unique peptides identified. Only proteins that were identified in all four biological replicates are shown. (C to E) Fraction of CD33 mRNA transcripts expressed as D2-CD33 (C), full-length CD33 (D), and D2-CD33 (E) mRNA levels measured by RT-qPCR in HeLa cells coexpressing PTBP1, SRSF1, or MATR3 with a CD33 minigene containing either rs12459419^C^ or rs12459419^T^, normalized to cells transfected with the wild-type T-allele minigene. (F) Fraction of CD33 mRNA transcripts expressed as D2-CD33 measured by RT-qPCR in HeLa cells expressing a CD33 minigene containing either the endogenous or an extended polypyrimidine (pY) tract in the presence of rs12459419^C^ or rs12459419^T^, normalized to wild-type cells containing rs12459419^T^. Error bars indicate means plus the SEM. A one-way ANOVA was performed. *, *P* ≤ 0.05; **, *P* ≤ 0.01; ****, *P* ≤ 0.0001; ns, not significant. *n* = 3 biological replicates for each plot, except for the SRSF1 isoform 1 sample in panels C and E, where *n* = 2 due to levels of D2-CD33 being below the limit of detection in one of the biological replicates.

The results of our siRNA screening, cDNA overexpression, and ASO scanning gave us insight into factors acting at the 3′ end of exon 2 that impacted alternative splicing. Given that the AD-associated rs12459419 SNP is located at the 5′ end of exon 2, we wanted to more directly examine potential splicing regulators affected by the rs12459419 genotype. Given the ability of mass spectrometry to identify a wider range of proteins interacting with CD33 pre-mRNAs, we developed an RNA pulldown-SILAC (stable isotope labeling with amino acids in cell culture) mass spectrometry analysis to quantitatively survey SNP-dependent proximal alterations of RNA-to-protein interactions. In short, synthetic 50-mer desthiobiotinylated RNA fragments containing the rs12459419^T^ or rs12459419^C^ SNP with surrounding CD33 gene sequence were coupled to streptavidin magnetic beads and incubated with lysates from SILAC-labeled THP1 cells. We aimed to capture relevant RNA-binding proteins and splicing components during the incubation of the myeloid cell lysates with the 50-mer CD33 RNA oligonucleotides. After washing away unbound factors, magnetic capture with streptavidin beads pulled down the desthiobiotinylated RNA fragments. rs12459419^T^ and rs12459419^C^ containing RNA fragment pulldowns were then combined so that SILAC heavy and SILAC light bound proteins were jointly eluted from beads before undergoing trypsin-digestion and quantitative analysis using liquid chromatography-tandem mass spectrometry (LC-MS/MS) (Fig. 6A). Forward and reverse SILAC experiments were each performed twice, and the ratio between protein abundance in rs12459419^C^ and rs12459419^T^ pulldown samples was calculated (Fig. 6B). Using a threshold of at least three unique tryptic fragments as a cutoff to positively include a protein as being present in either sample set, we utilized standard proteomic methodologies (Proteome Discoverer 2 [see Materials and Methods]) to calculate the fold enrichment of proteins between sample sets (see the supplemental material). Only proteins that were observed in all four biological replicates were included. Notably, Matrin3 (MATR3) and polypyrimidine tract-binding proteins 1 and 3 (PTBP1 and PTBP3) were >4-fold enriched in the rs12459419^C^ RNA pulldown (Fig. 6B, shown in red), whereas replication protein A2 (RPA2) and SRSF10 were >4-fold enriched in pulldowns of RNA fragments carrying the rs12459419^T^ allele (Fig. 6B, shown in blue).

Based on the number of unique peptides identified in the mass spectrometry, as well as their established role in pre-mRNA splicing, we chose to functionally interrogate MATR3 and PTBP1 as candidate factors with RNA-binding impacted by the rs12459419 genotype. PTBP1 is known to promote both selective exon inclusion and exon skipping events in pre-mRNA splicing (34, 35). Interestingly, MATR3 is a nuclear matrix protein that has been characterized as a binding partner of PTBP1. MATR3 alters splicing by enhancing or antagonizing the effect of PTBP1 depending on the context but can also affect alternative splicing independent of PTBP1 (36).

To test the functional effects of MATR3 and PTBP1 on CD33 exon 2 splicing, we cotransfected expression constructs containing these cDNAs along with our CD33 splicing constructs (carrying either rs12459419^C^ or rs12459419^T^ SNPs) in HeLa cells. Controls included in the assay were the coexpression of the CD33 intron-containing minigenes with SRSF1 isoform 1 (ASF-1) or isoform 3 (ASF-3), which are known to be a splicing enhancer and an inhibitor, respectively (37). Whereas the overexpression of SRSF1 isoform 3 in HeLa cells did not significantly alter CD33 exon 2 skipping, overexpression of SRSF1 isoform 1 boosted exon 2 inclusion and diminished D2-CD33 mRNA expression (Fig. 6C to E). Similarly, overexpression of PTBP1 reduced the level of D2-CD33 transcripts, as well as the fraction of total CD33 expressed as D2-CD33 by >11-fold (Fig. 6C and E). However, we found that the coexpression version of the assays exhibited higher variability than previously observed and that the amount of full-length CD33 trended higher but did not significantly increase between PTBP1-overexpressing and empty vector-transfected cells (Fig. 6D). In the case of MATR3 overexpression, only cotransfection with rs12459419^C^ containing CD33 splicing constructs yielded a significant reduction in the fraction of D2-CD33 (Fig. 6C), but a concordant C-allele-specific increase in full-length CD33 mRNA was not observed (Fig. 6D). It should be noted that while there was a trend toward a higher fold change in full-length CD33 mRNA from C-allele constructs coexpressed with MATR3, the higher-than-expected variability made interpretation of the result not straightforward.

PTBP1 is known to bind polypyrimidine (Py) tracts that are part of canonical splice junctions (38). Given that our synthetic RNA fragments used in the pulldown experiments included the complete upstream Py tract, we hypothesized that this sequence enhanced the binding of PTBP1 to the intron 1-exon 2 splice junction. Furthermore, the exon 2 proximal Py tract is only separated by 7 bp from the rs12459419 SNP, and this therefore may explain the genotype-dependent binding enrichment differences observed in our SILAC-MS/MS experiments (Fig. 6B). We therefore wondered whether extending the Py tract would compensate for the potentially weaker rs12459419^T^-mediated interaction with the CD33 exon 2 splice junction. Upon extending the Py tract length to test this question, we observed the proportion of D2-CD33 mRNA was greatly reduced with both C- and T-allele splicing assays, and the difference was no longer significant (Fig. 6F). Taken together, our quantitative SILAC experiments, overexpression of PTBP1 in the splicing assay, and extension of the upstream Py tract indicate that PTBP1 interaction adjacent to the rs12459419 SNP promotes the recognition of the intron 1-exon 2 splice junction during CD33 pre-mRNA processing.

## DISCUSSION

With the discovery and replication of AD protective genetic variants associated with the CD33 gene came efforts to understand the molecular mechanism underpinning the phenomenon. While other AD GWAS candidate genes have established causality with coding variants or SNPs that drive alterations in gene expression, the biology involved in CD33’s impact on AD risk has been attributed to a splicing phenotype. Profiling of human tissues led to the following observations: (i) the CD33 GWAS SNP correlated to elevated levels of CD33 mRNA isoforms lacking exon 2, (ii) this effect was dose dependent with the allelic genotype of rs3865444, and (iii) the rs12459419 SNP was likely the cause of the splicing effect due to its coinheritance with the GWAS SNP and functional impact in models of CD33 splicing.

Here, we find that the splicing regulators SRSF1 and PTBP1 act as splicing enhancers to increase exon 2 inclusion in the mature CD33 mRNA. Importantly, by measuring the impact of the rs12459419 SNP genotype on RNA-binding factors with quantitative proteomics, we identified a number of potential proteins that may contribute to the alternative splicing of CD33 (see the supplemental material). Our follow-up experiments that focused on known splicing mediators within the proteomics data set illustrate that the rs12459419^T^ SNP impaired binding of PTBP1 to the intron 1-exon 2 splice junction and functionally enhanced D2-CD33 isoform mRNA expression. Further directed binding experiments will help clarify whether PTBP1 is the sole determinant of the SNP-dependent effect on exon 2 inclusion. Our data point to a mechanism where PTBP1 is acting as a splice junction recognition enhancer. We anticipate that orthogonal biophysical analysis will be helpful in determining how the rs12459419^T^ SNP impacts PTBP1 affinity for the CD33 pre-mRNA.

Previous studies of PTBP1 have implicated the protein in several aspects of mRNA metabolism, including splicing regulation (34). PTBP1 possesses four RRMs that can bind to the RNA, with recognition of a core CU motif by RRM1, -2, and -3 (39–41). In addition, RRM2 can interact with proteins containing the PTB RRM2 interacting motif (42). One of PTB’s binding partners is MATR3, and both proteins were found to coregulate a set of splicing events in either the same or opposite direction (36). The observation that both PTBP1 and MATR3 bind more selectively to the CD33 RNA fragment carrying the 12459419^C^ allele (Fig. 6B) suggests that a similar coregulatory process might affect CD33 exon 2 splicing. Also, MATR3 seems to have some effects on CD33 exon 2 splicing (Fig. 6C to E). Although the effects of MATR3 are not significant in all cases and not as pronounced compared to PTBP1, it is expected that multiple factors act on CD33 exon 2 splicing since alternative splicing events are known to be controlled by the dynamic interplay between numerous splicing regulators with the pre-mRNA (20). Therefore, more rigorous studies of the interactions between PTBP1 and MATR3 in the context of CD33 splicing are warranted.

**FIG 7:**
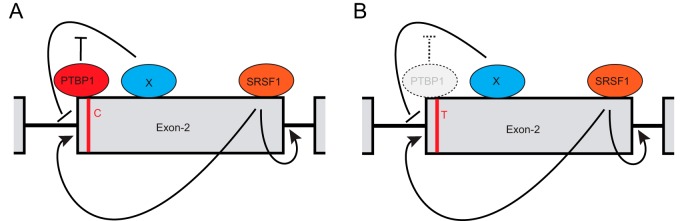
Proposed model for CD33 exon 2 splicing regulation. We propose that SRSF1 binding near the 3′ end of exon 2 enhances exon 2 inclusion in the CD33 pre-mRNA, regardless of the rs12459419 SNP genotype. At the same time, PTBP1 binding to the CD33 pre-mRNA also enhances exon 2 inclusion, potentially by antagonizing the action of other splicing regulators that functionally inhibit splice junction recognition. The rs12459419^T^ SNP reduces the binding of PTBP1 to the proximal Py tract, thus leading to elevated exon 2 skipping.

In most cases, PTBP1 acts as a repressor of exon inclusion (34) by competing with U2AF65 for binding to the polypyrimidine tract (43, 44). PTBP1 can also obstruct exon definition (45, 46) and interfere with intron definition (47, 48). However, genome-wide transcriptome analyses showed that PTBP1 can also promote exon inclusion (35, 49–51). For example, PTBP1 has been shown to counteract SRSF9-mediated splicing repressor activity and thereby stimulated exon inclusion (52). Interestingly, the RNA fragment we used for the RNA pulldown includes the complete polypyrimidine tract that is located in the intron upstream of exon 2. Moreover, PTBP1 has also been shown to bind to short RNA elements such as UCCU that are adjacent to non-pyrimidine-rich RNA sequences. The UCC and UCCU RNA elements are one of the top-scoring RNA motifs in a PTBP-CLIP experiment performed in HeLa cells (35). These data support the prediction that PTBP1 binds when rs12459419^C^ is present but not when rs124594149^T^ is present, which corroborates our RNA pulldown findings where PTBP1 was >4-fold enriched in the rs12459419^C^ sample. We propose that in the CD33 pre-mRNA, PTBP1 inhibits splicing repressors acting in *trans* and thereby stimulates CD33 exon 2 inclusion. In the case of the AD-associated rs12459419^T^ allele, reduced binding of PTBP1 to the CD33 exon 2 pre-mRNA would allow a competing splicing inhibitor to increase exon 2 skipping (Fig. 7).

In contrast to PTBP1, SRSF1 interaction with the CD33 RNA was not affected by the AD risk-associated SNP at the 5′ end of exon 2. Interestingly, disrupting or blocking an identified exonic splicing enhancer sequence that overlaps with a predicted SRSF1 binding element at the opposite end of exon 2, hampered exon 2 inclusion. SRSF1 is a prototypical SR protein that functions in constitutive and alternative splicing (53). Mechanistically, SRSF1 binds purine-rich pre-mRNA elements and simultaneously interacts with other splicing components through a C-terminal domain that is rich in arginine and serine residues (RS domain). Hence, upon binding of SRSF1 to the pre-mRNA, SRSF1 aids in the recognition of the 5′ splice site by recruiting the U1 small nuclear ribonucleoprotein (snRNP U1) (54). In addition to directing the U1 snRNP, SRSF1 also binds to the U2 snRNP, as well as U2AF35, and thereby pairs the correct 5′ and 3′ splice sites, which is a prerequisite for the correct excision of introns while avoiding exon skipping events (55, 56). Interestingly, it has been shown that when competing 5′ splice sites exist, SRSF1 elicits concentration-dependent changes in 5′ splice site selection and thereby affects alternative splicing (53, 57). Therefore, even though our functional genomic and proteomic profiling did not reveal differential interaction of SRSF1 in the RNA region proximal to the rs12459419 SNP, our discovery sheds light on higher-order regulation of the splicing machinery that leads to the two major CD33 isoforms.

Strikingly, the 381-nucleotide CD33 exon 2 contains four purine-rich RNA stretches that are putative SRSF1 binding sites. It is therefore not surprising that manipulating SRSF1 levels changes CD33 exon 2 splicing. Moreover, a positional bias toward the 5′ splice site has been related to the role of SRSF1 as an activator of exon inclusion, whereas binding near the 3′ splice site promoted either exon skipping or inclusion (58). These characteristics correlate with our finding that disrupting an SRSF1 binding site near the 3′ end, but not the 5′ end, of exon 2 stimulates the incorporation of CD33 exon 2. We observed a specific reduction in the proportion of D2-CD33 mRNA relative to full-length CD33 mRNA upon overexpressing SRSF1. Concordantly, SRSF1 knockdown significantly increased D2-CD33 relative to full-length CD33 transcripts. With our combined SRSF1 data set, we propose a model in which binding of SRSF1 near the 3′ end of exon 2 stimulates exon 2 inclusion into the processed CD33 transcript (Fig. 7).

The inability of the protein encoded by the D2-CD33 mRNAs to bind sialic acids and suppress microglia, resulting in increased phagocytic capacity of amyloid plaques, is one mechanism proposed by which the causal rs12459419 SNP modifies AD risk. Given that the ITIM-bearing Siglec family receptors such as CD33 can antagonize ITAM-mediated signals originating from receptors such as TREM2 (14, 59, 60), the molecular implications of CD33 loss of function may be a lower threshold for microglia to sense disruptions of CNS homeostasis. However, D2-CD33 might hold functions that are independent of exon 2-mediated sialic acid binding. One example of this is a report that complement component 1q (C1q) could bind to CD33 C_2_ domain in both full-length and D2-CD33 receptors to activate its inhibitory motifs (61). In addition, D2-CD33, in contrast to full-length CD33, has been shown to accumulate in peroxisomes, but whether CD33 has a peroxisomal function remains unknown (62). Our profiling of expressed CD33 isoforms by flow cytometry confirms that D2-CD33 proteins are less represented in the population of cell surface CD33 compared to full-length isoforms, which could be due to differential localization or altered stability (63). Both scenarios bolster the view of D2-CD33 as a loss-of-function variant from the perspective of ITIM-to-ITAM cross-regulation. As to the implication of how to phenocopy the CD33 biology as illuminated by AD genetics, it appears desirable to increase CD33 exon 2 skipping as a means to lower IgV domain-containing CD33 levels. We have identified SRSF1 and PTBP1 as enhancers of exon 2 inclusion. Thus, our data suggest that preventing the binding of SRSF1 and PTBP1 to the CD33 exon 2 pre-mRNA with either an ASO or small molecule might be a potential avenue to achieve a therapeutic effect on microglia in the central nervous system.

## MATERIALS AND METHODS

### DNA constructs.

The following constructs have been described: pCMV6-SRSF2-myc-DDK (Origene, RC209842, NM_003016), pCMV6-SRSF1-myc-DDK (Origene, RC201636, NM_006924), pCDNA3.1/Zeo(+) (Thermo Fisher Scientific, V86020), pCMV-3TAG-1a-PTBP1 (GenScript, clone OHu15891C, NM_002819.4), pCMV-3TAG-1a-MATR3 (GenScript, clone OHu17749C, NM_018834.5), pCMV-3TAG-1a-SRSF1-isoform 1 (GenScript, clone OHu24158C, NM_006924.4), and pCMV-3TAG-1a-SRSF1-isoform 3 (GenScript, clone OHu23760C, NM_001078166.1). All CD33 expression vectors were created by inserting CD33 into pCDNA3.1/Zeo(+) using a PCR based strategy (sequences were deposited into Addgene, see Table 1 ). The rs12459419 C→T nucleotide change was accomplished by using the Q5 site-directed mutagenesis kit (New England Biolabs, EO554S). Using the same strategy, mutations were made in the putative SRSF1 binding sites and the MOE-ASO targeting site (Table 2).

**TABLE 1:** Plasmid sequence links and usage key

Plasmid name, URL for sequence data	Figure(s)
pCD33_Exon1-7_minus introns_T, https://www.addgene.org/vector-database/10578	1
pCD33_Exon1-7_minus introns_C, https://www.addgene.org/vector-database/10579	1
pCD33_Exon1-7_plus introns_T, https://www.addgene.org/vector-database/10580	1, 3D to F, and 5E and F
pCD33_Exon1-7_plus introns_C, https://www.addgene.org/vector-database/10581	1, 3D to F, and 5E and F
pCD33_Exon1-4_plus introns_T, https://www.addgene.org/vector-database/10582	3A and B, 5A to D, and 6C to E
pCD33_Exon1-4_plus introns_C, https://www.addgene.org/vector-database/10583	3A and B, 5A to D, and 6C to E
pCD33_Exon1-7_plus introns_T_Extended pY, https://www.addgene.org/vector-database/10584	6F
pCD33_Exon1-7_plus introns_C_Extended pY, https://www.addgene.org/vector-database/10585	6F

**TABLE 2:** Mutation key for splicing constructs

Mutant	Nucleotides^*a*^	Mutation	Location
1	53/54	GA→TT	Intron 1
2	157/158	AG→TT	Exon 2
3	159/160	GA→TT	Exon 2
4	312/313	GA→TT	Exon 2
5	315/316	GA→TT	Exon 2
6	387/388	AG→TT	Exon 2
7	390/391	AG→TT	Exon 2
8	424/425	AG→TT	Exon 2
9	426/427	AG→TT	Exon 2
10	429/430	GG→TT	Exon 2
11	431–433	AAG→TTC	Exon 2
12	436–440	CCAAA→TTGGG	Exon 2
13	443–445	CAG→TTC	Exon 2

a First nucleotide of start codon = 1 (NC_000019.10).

### Cell lines. (i) CD33 splicing-reporter K562 cells.

By using CRISPR/Cas9 genome editing methodologies with the sgRNA (5′-TGGAGAGTCCCTGGATATAA-3′) and a plasmid donor, the P2A-NanoLuc-PEST-pA-LoxP-Neo-LoxP was inserted in frame with the third exon of CD33 in one allele of K562 (ATCC CCL-243) cells (ATCCCTGGCACTCTAGAACCC [P2A-NanoLuc-PEST-pA-LoxP flanked selection marker] GGCCACTCCAAAAACCTGAC) (Fig. 2A). Silent mutations were introduced to prevent recutting of the edited allele (TCCAGG→AGTCGC). In addition, two stop codons in exon 2 were created (TCAAGAA→TTAATAA and rs12459419: T→C). Generation of the cell line was performed by Horizon Discovery. Validation of genome editing and reporter expression was performed with comprehensive Sanger sequencing over the targeted genomic DNA region in addition to Illumina TruSeq whole-transcriptome RNA sequencing.

### (ii) THP1 CD33 KO cells.

CD33 knockout (KO) cells were created by transfecting the sgRNA (5′-TTGGGTTCTGTGGAACATCT-3′ and 5′-CCTCACTAGACTTGACCCAC-3′), together with Cas9-mRNA (Invitrogen, catalog no. 29378) and eGFP-mRNA (Trilink, L6101), into low-passage-number THP1 (ATCC TIB-202) cells using Lipofectamine MessengerMAX (Thermo Fisher Scientific, LMRNA003) according to the manufacturer’s protocol. Green fluorescent protein-positive cells were sorted into the wells of a 96-well plate using the Sony LE-SH800ZFP cell sorter. The clones were then split into a 96-well plate and a 24-well plate for genomic analysis and Western blot analysis, respectively, to detect CD33 KO clones, and all alleles were confirmed to contain nonsense mutations with miSeq of genomic DNA PCR amplicons.

### Cell culture and transfection.

K562 and THP1 cells were cultured in filter sterilized RPMI 1640 (Gibco, 21870-076) containing 10% heat-inactivated, low-endotoxin FBS (Gibco, catalog no. 10082139) and 2 mM GlutaMAX supplement (Gibco, catalog no. 35050061). HeLa cells (ATCC CCL2) were cultured in filter-sterilized Dulbecco modified Eagle medium–F-12 (Gibco, catalog no. 11320033) with 2 mM GlutaMAX supplement and 10% fetal bovine serum (FBS).

### (i) Transfection of HeLa cells with cDNAs using FugeneHD transfection reagent.

Cells were plated in a tissue culture (TC) treated 24-well plate 1 to 2 days prior to transfection. When cultures reached 80% confluence, cells were transfected with FugeneHD transfection reagent (Promega, E2311) according to the manufacturer’s protocol. For each transfection, 1.5 μl of FugeneHD and 0.5 μg of CD33 minigene (Fig. 1 and 5) or 50 ng of CD33 minigene plus 450 ng of splicing factor (Fig. 6C to E and Fig. 3A, B, and D to F) were used.

### (ii) Transfection of HeLa cells with cDNAs using the Neon transfection system.

A total of 5 × 10^4^ cells were electroporated with 0.5 μg of plasmid DNA per 10-μl sample, using two pulses of 35 ms at 1,005 V according to the manufacturer’s instructions. The cells were then immediately added to 500 μl of culture medium in a 24-well TC-treated plate and allowed to attach.

### (iii) Reverse transfection of K562 cells with siRNAs using Lipofectamine RNAiMAX.

A 2-μl portion of a 1 μM siRNA solution in nuclease-free water was transferred from a 384 source plate into a 384 assay plate (Corning, catalog no. 3765). Each plate contained replicate siRNAs against NanoLuc (sense, GGAUUGUCCUGAGCGGUGATT; antisense, UCACCGCUCAGGACAAUCCTT), custom Select siRNA (Ambion), and replicates of a nontargeting control (NTC; Thermo Fisher Scientific, Ambion Silencer Select Negative Control 2, catalog no. 4390846), and replicate wells were left without any siRNA (reserved for media only mock-transfected cells). Only a single siRNA reagent was placed in each well to avoid complex off-target effects. See the supplemental material for sequences and reagent IDs. A master mix containing 19.88 μl of Opti-MEM and 0.12 μl of Lipofectamine RNAiMAX (Thermo Fisher Scientific, catalog no. 13778150) per sample was made, followed by incubation for 5 min at room temperature. Then, 20 μl of this mix was added to the siRNAs, followed by incubation for 30 min at room temperature. Meanwhile, cells from a low-passage-number culture of the K562 or K562 splicing reporter line in the linear growth stage were pelleted for 5 min at 200 × *g* and resuspended in RPMI 1640 medium (Gibco, catalog no. 21870-076) containing 20% heat-inactivated, low-endotoxin FBS (Gibco, catalog no. 10082139) and 2 mM GlutaMAX supplement (Gibco, catalog no. 35050061) to a density of 1 × 10^5^ cells ml^−1^. Next, 20 μl of the cell suspension was dispensed into each well containing the DNA/lipid mix by using an automated peristaltic liquid handler. The final concentration of siRNA reagent in each well was 50 nM in a total culture volume of 40 μl per well. Assay plates were then incubated in a tissue culture incubator with 5% CO_2_ and 95% humidity for 72 h.

### (iv) Reverse transfection of K562 cells with MOE-ASOs using Lipofectamine 2000.

Similar to siRNA screening, MOE-ASO transfection for screening was performed in 384-well assay plates (Corning, catalog no. 3765). The transfection process began with 0.8-μl portions of MOE-ASO reagents (250 pmol μl^−1^) (see the supplemental material) in nuclease-free water being added to 19.2 μl of Opti-MEM per sample. MOE-ASOs had a phosphorothioate backbone, and all nucleotides were MOE modified, except the 3′ base. The MOE-ASOs were made by Dharmacon/Horizon Discovery and IDT-DNA Technologies. In polypropylene tubes, a master mix containing 1.3 μl of Lipofectamine 2000 (Thermo Fisher Scientific, catalog no. 11668019) and 18.7 μl of Opti-MEM per sample was mixed. After 5 min of incubation at room temperature, 20 μl of the Lipofectamine/Opti-MEM master mix was added to the 20 μl of the MOE-ASO/Opti-MEM mix in each well of the assay plates using an automated pipetting system, and then the plates were incubated at room temperature for 30 min. Meanwhile, K562 cells containing the CD33 splicing reporter were pelleted for 5 min at 200 × *g* in a centrifuge. Subsequently, 10 μl of a 2 × 10^5^ ml^−1^ cell suspension was plated into each well of a 384-well plate using an automated peristaltic reagent dispenser. To initiate transfection, for each ASO three wells were transfected by adding 10 μl of the ASO/Lipofectamine/Opti-MEM mixture to each well.

### (v) Transfection of THP1 cells with siRNAs or MOE-ASOs using the Neon transfection system.

A total of 3 × 10^5^ cells were electroporated with 60 nM solutions of siRNA or 10 μM MOE-ASO reagent per 10-μl Neon tip (Invitrogen, MPK1025), using two pulses of 20 ms at 1,200 V according to the manufacturer’s instructions. Cells were then immediately added to 500  μl of culture medium in 24-well plates.

### RT-PCR and RT-qPCR.

At 24 to 48 h posttransfection, mRNA from cells was isolated by using an RNeasy Plus minikit (Qiagen, catalog no. 74134) and QIAshredder spin columns (Qiagen, catalog no. 79654). The RNA concentration was determined with a NanoDrop 2000 (Thermo Fisher Scientific, ND-2000). For each sample, cDNA was generated by using iScript reverse transcription supermix (Bio-Rad, catalog no. 1708841). For RT-qPCR, SSO Advanced Universal Probes Supermix (Bio-Rad, catalog no. 1725282) was used to carry out the RT-qPCR in a 10-μl reaction according to the manufacturer’s instructions on a Bio-Rad CFX384 RT-qPCR machine. The following probes and primers were used: CD33-exon-2 present (probe, 56-FAM/TGCATGTGA/ZEN/CAGACTTGACCCACA/3IABkFQ; Fw-primer, TTCGGATGGAGAGAGGAAGTA; Rv-primer, GTGCCAGGGATGAGGATTT) and CD33-exon-2 skipped (probe, HEX/TGTGGGCAG/ZEN/ACTTGACCCACAG/3IABkFQ; Fw-primer, CGCTGCTGCTACTGCTG; Rv-primer, TTCTAGAGTGCCAGGGATGA [IDT-DNA Technologies]). We also used the following TaqMan assays: SRSF1, Hs00199471_m1; SRSF2, Hs01089823_m1; GAPDH, Hs02758991_g1; and hnRNPH1, Hs00800662. The CD33 primer/probe sets were validated by mixing gBlocks (IDT-DNA Technologies) gene fragments corresponding to exons 1, 2, and 4 or exons 1, 2, 3, and 4 of human CD33 at different ratios. TaqMan assays were purchased from Thermo Fisher Scientific. For the RT-PCRs analyzed on a 1.2% agarose gel, Fw-primer (CTCAGACATGCCGCTGCT) and Rv-primer (TTGAGCTGGATGGTTCTCTCCG) were used.

### Fluorescence-activated cell sorting analysis.

Cells were collected in 200 μl of culture medium and transferred to a deep V-bottom 96-well plate (polypropylene). Subsequently, cells were pelleted by centrifugation at 300 × *g* for 3 min and resuspended in 100 μl of ice-cold phosphate-buffered saline (PBS; Gibco, catalog no. 14190250) supplemented with 1% FBS and the corresponding antibodies (1:5 for CD33 antibodies and 1:20 for CD11b). The antibodies used were as follows: phycoerythrin (PE)-conjugated mouse anti-human CD33 clone WM-53 (BD Biosciences), fluorescein isothiocyanate (FITC)-conjugated mouse anti-human CD33 clone him3-4 (BD Biosciences, catalog no. 555626), and Alexa Fluor 488-conjugated anti-human CD11b clone ICRF44 (BioLegend, catalog no. 301317). Upon incubation at 4°C for 20 min, the cells were washed three times in PBS plus 1% FBS and analyzed with MacsQuant VYB (Miltenyi Biotec, 130-096-116).

### Statistical analysis and software used.

FlowJo v.10.1 was used to analyze the fluorescence-activated cell sorting data. RT-qPCR data were analyzed using Bio-Rad CFX manager 3.1. mRNA levels of D2-CD33, full-length CD33, hnRNPH1, SRSF1, and SRSF2 were normalized to GAPDH (glyceraldehyde-3-phosphate dehydrogenase) levels to obtain Δ*C_T_* values (64). Subsequently, 2^–Δ^*^CT^* values were normalized to control cells under each condition (NTC, empty vector, or wild-type T-allele) (Fig. 2D, E, and I; Fig. 3A, C, and H; Fig. 4E; Fig. 5C; and Fig. 6D and E). The fraction of CD33 expressed as D2-CD33 was calculated as D2-CD33/(D2-CD33 + full-length CD33) using the 2^–Δ^*^CT^* values as described above. Subsequently, values were normalized to control samples under each condition (NTC, empty vector, or wild-type T-allele) (Fig. 2G and H; Fig. 3B and G; Fig. 4C and D; Fig. 5B and D; and Fig. 6C and F). GraphPad Prism 7 was used to generate graphs/plots and perform statistical analysis (two-tailed unpaired *t* test [Fig. 1; Fig. 4D, E, and H; Fig. 5F] and one-way analysis of variance [ANOVA] [Fig. 2, Fig. 3, Fig. 4B and C, Fig. 5B to D, Fig. 6]). All LC-MS/MS runs were analyzed using Sequest algorithm (Sequest HT; Thermo Scientific) within Proteome Discoverer 2.1 (Thermo Scientific) against the Swiss-Prot human database (May 2017, 42,085 entries). Genedata Screener was used to analyze luminescence levels in the CD33 splicing reporter cell line. Significance is indicated in the figures.

### Luciferase assays.

For siRNA and MOE ASO cell-based screening, NanoLuc reporter expression was determined by using a Nano-Glo luciferase assay system (Promega, N1120), whereas CellTiter-Glo (Promega, G7572) was used to determine the number of viable cells according to the manufacturer’s protocols. Nano-Glo substrate solution was prepared according to manufacturer’s recommended protocols and at the assay endpoint. A 40-μl portion of Nano-Glo was dispensed into each well of the 384-well plates using an automated peristaltic microplate dispenser, followed by incubation at room temperature for 10 min. Similarly, 40 μl of CellTiter-Glo solution was dispensed into the wells of cell viability plates. The luminescent signal from each well of the 384-well assay plate was read using an Envision apparatus (Perkin-Elmer).

### SILAC experiment. (i) Cell culture.

THP1 cells were expanded in filter sterilized RPMI 1640 SILAC medium (Thermo Fisher Scientific, catalog no. 88365) supplemented with 10% dialyzed fetal calf serum (Thermo Fisher Scientific, catalog no. 26400044), 100 U ml^−1^ penicillin and 100 μg ml^−1^ streptomycin (Thermo Fisher Scientific, catalog no. 15140122), 2 mM l-glutamine (Thermo Fisher Scientific, catalog no. 25030081), 1× MEM nonessential amino acid solution (Thermo Fisher Scientific, catalog no. 11140050), and 25 mM HEPES (Thermo Fisher Scientific, catalog no. 15630080). “Heavy” medium was supplemented with 50 mg of l-lysine–2HCl (^13^C_6_, 99%; ^15^N_2_, 99%) (Cambridge Isotope Laboratories, CNLM-291-H-0.1) and 50 mg of l-arginine–HCl (^13^C_6_, 99%; ^15^N_4_, 99%) (Cambridge Isotope Laboratories, CNLM-539-H-0.1). “Light” medium was supplemented with 50 mg of l-lysine:2HCl (unlabeled) (Cambridge Isotope Laboratories, ULM-8766-0.1) and 50 mg of l-arginine–HCl (unlabeled) (Cambridge Isotope Laboratories, ULM-8347-0.1). After thawing, the cells were passaged at least three times before being used in experiments.

### (ii) Cell lysis.

Cells were washed twice with ice-cold PBS (Gibco, catalog no. 14190250). Per sample, 20 million cells were lysed for 1 h at 4°C in 1.5 ml of ice-cold NP-40 cell lysis buffer (Thermo Fisher Scientific, FNN0021) supplemented with protease inhibitors (Sigma, catalog numbers P8340, P5726, and P0044). The cell lysate was centrifuged at 4°C at 17.900 × *g*, the supernatant was transferred to a new tube, and the protein concentration was measured using the Pierce BCA protein assay kit (Thermo Fisher Scientific, catalog no. 23225). All samples were diluted to the same protein concentration, and Tween 20 was added to a final concentration of 0.05%.

### (iii) Conjugation of RNA to streptavidin beads.

Per sample, 30 μl of MyOne streptavidin C1 Dynabeads (Thermo Fisher Scientific, catalog no. 65001) was washed with binding and washing buffer (B&W buffer; 5 mM Tris-HCl [pH 7.5], 0.5 mM EDTA, 2 M NaCl). After the last wash, the beads were incubated twice in buffer A (0.1 M NaOH, 0.05 M NaCl) for 2 min, after which the beads were washed twice with buffer B (0.1 M NaCl) and three times with B&W buffer.

### (iv) Pulldown of proteins interacting with CD33 RNA oligonucleotides.

Beads were resuspended in 200 μl of B&W buffer containing 600 pmol of desthiobiotinylated RNA (synthesized by IDT-DNA Technologies). After incubation for 15 min at room temperature, 600 pmol of d-desthiobiotin (Sigma-Aldrich, D1411-500MG) was added to each sample, and the samples were incubated for another 10 min. Beads were washed three times with B&W buffer, and lysates were added to the beads. For the forward SILAC experiment, rs12459419^T^-containing desthiobiotinylated RNA (see sequences below) was incubated with the “heavy” THP1 cell lysate, whereas rs12459419^C^-containing RNA fragments were incubated with the “light” THP1 cell lysate. After 1 h of incubation at 4°C with gentle rotation, the beads were washed three times with Tris-buffered saline with 0.05% Tween 20. After the third wash, beads incubated with heavy and light lysates were combined, and proteins were eluted for 10 min in 250 μl of NP-40 cell lysis buffer (Thermo Fisher Scientific, FNN0021) supplemented with protease inhibitors (Sigma, catalog numbers P8340, P5726, and P0044) and 16 mM biotin (B4501-1G). Proteins were precipitated by adding 1,750 μl of ice-cold 100% ethanol and stored at –80**°**C.

### (v) Proteomic sample preparation.

The next day, samples were pelleted for 30 min at 4°C and resuspended in 20 μl of 8 M urea (Invitrogen, ZU10001). Urea was diluted by adding 170 μl of 50 mM Tris (pH 8.0). Dithiothreitol (Sigma-Aldrich, D9163-1G) was added to a final concentration of 10 mM, and mixtures were incubated with rotation for 30 min. Subsequently, iodoacetamide (Sigma-Aldrich, I1149-5G) was added to a final concentration of 12.5 mM, and samples were incubated for 30 min at 37°C in the dark. To allow trypsin digestion, CaCl_2_ (Fluka BioChemica, 21098-500GR) was added to a final concentration of 1 mM CaCl_2_ together with 2 μg of trypsin (Promega, V5111). Samples were incubated overnight at 37°C with rotation. The next day, trifluoroacetic acid (TFA; Fisher Scientific, A116-50) was added to a final concentration of 2%, and samples were dried using a speed vacuum concentrator. Samples were dissolved in 5% TFA, desalted using a 10-μl ZipTip C18 (EMD Millipore, ZTC18S096), and eluted in 80% acetonitrile (Thermo Fisher Scientific, A955-500) and 0.1% formic acid (FA; Sigma-Aldrich, 56302-50ml).

### (vi) LC-MS/MS and data analysis.

After drying, the samples were resuspended in 10 μl of 0.1% FA, and 3 μl was analyzed on an Orbitrap Fusion Lumos mass spectrometer (Thermo Scientific) with an Easy Nanospray ion source (Thermo Scientific). Chromatographic separation of peptides was accomplished by using an Easy-nLC 1200 ultra-high-pressure liquid chromatography pump (Thermo Scientific). A 3-μl sample was analyzed on an Orbitrap Fusion Lumos mass spectrometer (Thermo Scientific). Mobile phases A and B were 0.1% FA in water (vol/vol) and 80/20/0.1% ACN/water/FA (vol/vol/vol), respectively. Peptides were loaded onto a reversed-phase trap (Acclaim PepMap; 2 cm, C_18_ resin 3 μm, 100 Å [Thermo Fisher Scientific]) with 100% mobile phase A and a series of Nanoflow gradients (flow rate at 250 nl/min) to backflush the trapped samples onto the nano-LC column (Easy Spray column, PepMap, 50 cm, C_18_ resin 2 μm, 100 Å [Thermo Fisher Scientific, catalog no. 03052574]) for separation. The nano-LC column was heated at 45°C to improve both chromatographic resolution and reproducibility. A 2-h gradient was used to achieve sufficient peptide separation. The optimized gradient profile was as follows: 5 to 40% B over 0 to 120 min; 40 to 95% B over 120 to 130 min; and isocratic at 95% B over 130 to 140 min. The Orbitrap Fusion Lumos mass spectrometer was operated using data-dependent acquisition (DDA) with a 3-s cycle time. MS^1^ scans had a resolution of 120,000, an automatic gain control (AGC) of 5e5, and a maximum ion injection time of 50 ms and were scanned from 375 to 1575 *m/z*; DDA HCD-MS^2^ scans had a resolution of 30,000, an AGC of 5e4, and a maximum injection time of 100 ms, with charge exclusion applied to unassigned and charge 5 and above. Previously interrogated precursors were excluded using a dynamic window (30 s ± 10 ppm). LC-MS/MS raw. files were processed using the Proteome Discoverer 2.1 (Thermo Scientific) applying Sequest HD for protein identification. The samples were searched against a nonredundant human UniProtSP database with carbamidomethylation (+57.021 Da) as a fixed modification and oxidation of methionines (+15.995 Da), heavy lysine (+8.014 Da), and heavy arginine (+10.008 Da) as variable modifications. The precursor mass tolerance was set to 10 ppm for the MS^1^, and a fragment mass tolerance to 0.02 Da with up to two missed cleavages was allowed. Final protein lists were filtered to only include peptides with a mass tolerance of <10 ppm and a false-positive rate at a protein level of less than <1%. The list of identified proteins was further filtered to exclude proteins with fewer than three unique peptides and proteins that were not found in all four biological replicates. The data are presented as mean SILAC ratios from the combined biological replicates (*n* = 2). The desthiobiotinylated RNA oligonucleotides used were C-allele (GAGCTGACCCTCGTTTCCCCACAGGGGCCCTGGCTATGGATCCAAATTTC) and T-allele (GAGCTGACCCTCGTTTCCCCACAGGGGTCCTGGCTATGGATCCAAATTTC) (the single difference between the two oligonucleotide sequences is underlined).

## Supplementary Material

Supplemental file 1
