# Supplementary material for: SRSF1 and PTBP1 Are trans-Acting Factors That Suppress the Formation of a CD33 Splicing Isoform Linked to Alzheimer’s Disease Risk
Source: Mol Cell Biol. 2019 Aug 27;39(18):e00568-18. doi: 10.1128/MCB.00568-18 (PMC6712934; doi:10.1128/MCB.00568-18)
Supplement: Supplemental file 1 [file MCB.00568-18-s0001.pdf]

Supplementary Table: Results of RNA-pulldown SIALC mass spectrometry experiment

This list encompasses proteins that bound to the CD33 exon-2 splice junction RNA fragments in all four biological replicates of the RNA-pulldown SILAC mass spectrometry experiment.

* Green shaded columns represent the protein ratios found in rs12459419T versus rs12459419C samples in the forward (Heavy/Light (rs12459419T/rs12459419C)) SILAC experiments whereas blue shaded conlumns indicates the reverse SILAC experiments (Heavy/Light (rs12459419C/rs12459419T)).

Accession	Gene Name	Description	Forward run 1 ratio*	Forward run 2 ratio*	Avg ratio [Forward runs]	% CV [Forward runs]	Log2 transformed ratio [Forward runs]	Reverse run 1 ratio*	Reverse run 2 ratio*	Avg ratio [Reverse Runs]	% CV [Reverse runs]	Log2 transformed ratio [Reverse runs]	Number of Unique Peptides	Number of Peptides	Number of PSMs	Number of Protein Unique Peptides	Number of Razor Peptides	emPAI	Score Sequest HT	Protein Coverage Sequest HT	Number of Peptides Sequest HT	Number of PSMs Sequest HT
P68133	ACTA1	Actin, alpha skeletal muscle OS	0,089	1,451	0,770	125,018	-0,377	1,240	0,877	1,058	24,275	0,082	6	18	216	0	0	109,53	400,86	51,98938992	18	216
P62736	ACTA2	Actin, aortic smooth muscle OS	0,040	1,351	0,695	133,286	-0,524	1,210	0,864	1,037	23,593	0,052	6	17	180	0	0	48,619	294,84	54,64190981	17	180
P60709	ACTB	Actin, cytoplasmic	0,307	3,887	2,097	120,696	1,068	1,050	0,826	0,938	16,886	-0,092	11	23	362	0	24	669,019	897,43	73,86666667	23	362
P68032	ACTC1	Actin, alpha cardiac muscle 1 OS	0,089	1,351	0,720	123,875	-0,474	1,050	0,877	0,963	12,723	-0,054	7	19	218	0	0	134,031	402,82	57,5596817	19	218
P63261	ACTG1	Actin, cytoplasmic 2	0,307	3,887	2,097	120,696	1,068	1,570	0,826	1,198	43,914	0,261	11	23	362	0	0	669,019	897,43	73,86666667	23	362
P63267	ACTG2	Actin, gamma-enteric smooth muscle OS	0,040	1,351	0,695	133,286	-0,524	0,980	0,864	0,922	8,896	-0,117	6	17	180	0	0	58,255	294,84	54,78723404	17	180
Q12904	AIMP1	Aminoacyl tRNA synthase complex-interacting multifunctional protein 1 O	0,944	1,060	1,002	8,233	0,003	0,913	0,670	0,792	21,649	-0,337	8	8	65	8	0	4,456	117,99	49,03846154	8	65
Q86V81	ALYREF	THO complex subunit 4 OS	0,852	0,804	0,828	4,042	-0,272	1,274	1,105	1,190	10,044	0,251	9	9	170	9	0	53,117	548,94	49,41634241	9	170
Q10567	AP1B1	AP-1 complex subunit beta-1 OS	0,528	0,713	0,621	21,039	-0,688	1,827	1,526	1,677	12,693	0,746	6	6	40	0	0	0,66	63,49	7,586933614	6	40
P63010	AP2B1	AP-2 complex subunit beta OS	0,564	0,654	0,609	10,491	-0,716	1,990	1,496	1,743	20,068	0,802	15	15	73	9	0	2,467	96,58	19,10352188	15	73
Q96CW1	AP2M1	AP-2 complex subunit mu OS	0,484	0,890	0,687	41,744	-0,541	1,708	1,458	1,583	11,147	0,662	3	3	27	3	0	0,487	65,87	14,02298851	3	27
Q12797	ASPH	Aspartyl/asparaginyl beta-hydroxylase OS	1,008	0,699	0,853	25,610	-0,229	0,932	0,825	0,879	8,559	-0,187	19	19	126	19	0	6,848	189,16	27,96833773	19	126
Q9Y224	C14orf166	UPF0568 protein C14orf166 OS	0,481	0,842	0,661	38,550	-0,597	2,253	2,321	2,287	2,092	1,194	7	7	67	7	0	4,275	152,08	38,52459016	7	67
P00918	CA2	Carbonic anhydrase 2	0,363	20,000	10,182	136,379	3,348	20,000	20,000	20,000	0,000	4,322	4	4	19	4	0	0,968	24,44	20,76923077	4	19
P0DP23	CALM1	Calmodulin-1 OS	0,696	1,311	1,004	43,321	0,006	1,410	0,994	1,202	24,449	0,266	6	6	97	6	0	49,119	225,14	71,81208054	6	97
Q01518	CAP1	Adenylyl cyclase-associated protein 1	0,282	11,308	5,795	134,539	2,535	8,240	4,166	6,203	46,444	2,633	18	18	89	17	0	6,055	130,88	45,47368421	18	89
Q14444	CAPRIN1	Caprin-1 OS	0,938	0,872	0,905	5,159	-0,145	1,193	0,963	1,078	15,091	0,108	16	16	210	16	0	30,623	539,08	23,83638928	16	210
Q13185	CBX3	Chromobox protein homolog 3 OS	1,222	1,806	1,514	27,257	0,598	1,389	0,915	1,152	29,119	0,204	3	3	22	2	0	2,162	49,37	23,49726776	3	22
P23528	CFL1	Cofilin-1 OS	0,473	2,425	1,449	95,276	0,535	1,903	1,449	1,676	19,158	0,745	7	7	66	4	0	25,102	148,61	62,04819277	7	66
Q9Y281	CFL2	Cofilin-2 OS	0,674	2,408	1,541	79,581	0,623	3,069	1,604	2,336	44,339	1,224	3	3	27	0	0	3,125	39,27	18,6746988	3	27
Q14011	CIRBP	Cold-inducible RNA-binding protein OS	0,563	0,374	0,469	28,465	-1,093	2,252	2,191	2,222	1,920	1,152	5	5	46	5	0	4,878	118,04	33,13953488	5	46
P09669	COX6C	Cytochrome c oxidase subunit 6C OS	1,005	0,893	0,949	8,395	-0,076	1,348	0,954	1,151	24,198	0,203	3	3	45	3	0	13,678	114,24	28	3	45
P08311	CTSG	Cathepsin G OS	0,911	0,415	0,663	52,900	-0,593	1,273	1,325	1,299	2,867	0,377	3	3	24	3	0	1,371	36,2	15,68627451	3	24
Q92499	DDX1	ATP-dependent RNA helicase DDX1 OS	0,487	0,459	0,473	4,186	-1,080	2,837	3,186	3,012	8,210	1,590	8	8	27	8	0	0,714	44,92	20,54054054	8	27
Q92841	DDX17	Probable ATP-dependent RNA helicase DDX17 OS	1,037	1,236	1,136	12,353	0,184	1,261	1,092	1,177	10,136	0,235	14	20	114	14	9	4,957	194,99	31,41289438	20	114
Q9NVP1	DDX18	ATP-dependent RNA helicase DDX18 OS	1,012	0,883	0,948	9,650	-0,078	1,239	0,967	1,103	17,413	0,142	9	9	68	9	0	1,966	88,17	19,40298507	9	68
P26196	DDX6	Probable ATP-dependent RNA helicase DDX6 OS	1,041	1,651	1,346	32,046	0,429	1,045	1,251	1,148	12,689	0,199	6	6	24	6	0	1,043	29,76	22,56728778	6	24
Q08211	DHX9	ATP-dependent RNA helicase A OS	1,266	1,226	1,246	2,269	0,318	0,825	0,771	0,798	4,756	-0,326	36	36	352	36	0	6,017	762,51	37,32283465	36	352
P68104	EEF1A1	Elongation factor 1-alpha 1 OS	0,665	2,697	1,681	85,470	0,749	1,540	1,926	1,733	15,765	0,793	13	13	98	0	0	9,965	204,78	49,78354978	13	98
Q5VTE0	EEF1A1P5	Putative elongation factor 1-alpha-like 3 OS	0,665	2,697	1,681	85,470	0,749	1,540	1,926	1,733	15,765	0,793	13	13	98	0	0	9,965	204,78	49,78354978	13	98
Q05639	EEF1A2	Elongation factor 1-alpha 2 OS	0,590	3,112	1,851	96,318	0,888	1,121	1,437	1,279	17,454	0,355	7	7	44	0	0	2,631	94,61	20,30237581	7	44
P47813	EIF1AX	Eukaryotic translation initiation factor 1A, X-chromosomal OS	1,483	1,734	1,608	11,058	0,685	0,918	0,807	0,862	9,062	-0,214	3	3	10	0	0	1,512	10	29,16666667	3	10
O14602	EIF1AY	Eukaryotic translation initiation factor 1A, Y-chromosomal OS	1,483	1,734	1,608	11,058	0,685	0,918	0,807	0,862	9,062	-0,214	3	3	10	0	0	1,512	10	29,16666667	3	10
P05198	EIF2S1	Eukaryotic translation initiation factor 2 subunit 1 OS	1,053	0,883	0,968	12,414	-0,046	0,858	0,614	0,736	23,469	-0,442	10	10	58	10	0	4,337	88,22	40	10	58
P20042	EIF2S2	Eukaryotic translation initiation factor 2 subunit 2 OS	1,144	1,252	1,198	6,393	0,261	1,189	0,592	0,891	47,352	-0,167	13	13	89	13	0	8,006	194	39,33933934	13	89
P41091	EIF2S3	Eukaryotic translation initiation factor 2 subunit 3 OS	1,065	1,227	1,146	10,037	0,197	1,014	0,719	0,867	24,073	-0,207	12	12	98	3	0	7,254	189,68	36,86440678	12	98
Q2VIR3	EIF2S3L	Putative eukaryotic translation initiation factor 2 subunit 3-like protein OS	1,065	1,227	1,146	10,037	0,197	1,077	0,733	0,905	26,826	-0,144	9	9	79	0	0	4,484	168,88	28,60169492	9	79
Q15717	ELAVL1	ELAV-like protein 1 OS	0,713	0,683	0,698	3,005	-0,518	1,535	1,477	1,506	2,723	0,591	15	15	201	14	0	113,505	545,18	45,09202454	15	201
P06733	ENO1	Alpha-enolase	0,064	7,248	3,656	138,933	1,870	8,814	13,574	11,194	30,066	3,485	7	7	24	6	0	1,043	21,95	25,34562212	7	24
P07814	EPRS	Bifunctional glutamate/proline--tRNA ligase OS	0,906	0,954	0,930	3,674	-0,104	0,900	0,671	0,785	20,649	-0,349	24	24	112	24	0	1,379	193,17	23,01587302	24	112
P15311	EZR	Ezrin OS	1,406	2,456	1,931	38,459	0,949	1,871	1,462	1,667	17,351	0,737	3	3	29	0	0	0,343	19,95	4,436860068	3	29
Q14331	FRG1	Protein FRG1 OS	1,151	1,351	1,251	11,284	0,323	1,150	0,919	1,035	15,744	0,049	6	6	40	6	0	4,179	45,69	18,99224806	6	40
P35637	FUS	RNA-binding protein FUS OS	1,180	1,088	1,134	5,717	0,181	0,550	0,641	0,595	10,848	-0,748	4	4	23	4	0	1,448	55,69	13,49809886	4	23
Q13283	G3BP1	Ras GTPase-activating protein-binding protein 1 OS	0,839	1,120	0,980	20,286	-0,030	1,204	1,003	1,103	12,884	0,142	17	18	203	17	0	73,989	461,75	56,43776824	18	203
Q9UN86	G3BP2	Ras GTPase-activating protein-binding protein 2 OS	0,805	1,694	1,250	50,272	0,321	1,239	1,044	1,142	12,098	0,191	18	19	271	18	2	33,145	595,17	45,43568465	19	271
P07305	H1F0	Histone H1.0 OS	0,436	0,377	0,407	10,147	-1,299	0,284	0,281	0,283	0,750	-1,822	6	6	127	6	0	176,828	281,06	30,92783505	6	127
Q92522	H1FX	Histone H1x OS	0,923	0,784	0,853	11,490	-0,229	1,045	1,015	1,030	2,082	0,043	4	4	52	4	0	4,179	113,83	15,96244131	4	52
P57053	H2BFS	Histone H2B type F-S OS	1,108	2,022	1,565	41,286	0,646	0,697	0,607	0,652	9,727	-0,617	4	4	47	0	0	3,329	95,02	34,92063492	4	47
P40939	HADHA	Trifunctional enzyme subunit alpha, mitochondrial OS	1,307	1,014	1,160	17,887	0,214	0,989	0,772	0,881	17,420	-0,183	10	10	53	10	0	1,268	82,38	20,44560944	10	53
P51858	HDGF	Hepatoma-derived growth factor OS	0,017	0,000	0,008	141,421	-6,907	0,007	0,000	0,003	141,421	-8,229	5	6	30	5	0	1,581	61,74	23,75	6	30
Q02539	HIST1H1A	Histone H1.1 OS	0,258	0,355	0,307	22,289	-1,705	0,162	0,145	0,154	7,831	-2,704	5	9	140	0	0	34,112	349,97	20	9	140
P16401	HIST1H1B	Histone H1.5 OS	0,600	0,627	0,614	3,073	-0,704	0,486	0,454	0,470	4,915	-1,089	11	15	234	11	0	202,092	530,03	29,20353982	15	234
P16403	HIST1H1C	Histone H1.2 OS	0,086	0,093	0,089	5,805	-3,485	0,084	0,076	0,080	7,381	-3,647	11	15	357	0	0	169,125	851,04	31,45539906	15	357
P16402	HIST1H1D	Histone H1.3 OS	0,086	0,093	0,090	6,057	-3,482	0,081	0,072	0,076	8,355	-3,715	12	16	395	0	4	241,446	971,67	30,76923077	16	395
P10412	HIST1H1E	Histone H1.4 OS	0,086	0,093	0,090	6,057	-3,482	0,081	0,072	0,076	8,355	-3,715	12	16	395	0	0	382,119	971,67	31,05022831	16	395
P22492	HIST1H1T	Histone H1t OS	0,258	0,355	0,307	22,289	-1,705	0,162	0,146	0,154	7,331	-2,696	5	5	125	0	0	20,544	312,09	8,695652174	5	125
P33778	HIST1H2BB	Histone H2B type 1-B OS	1,108	2,022	1,565	41,286	0,646	0,697	0,607	0,652	9,727	-0,617	3	3	46	0	0	2,511	95,02	27,77777778	3	46
P62807	HIST1H2BC	Histone H2B type 1-C/E/F/G/I OS	1,108	2,022	1,565	41,286	0,646	0,697	0,607	0,652	9,727	-0,617	4	4	47	0	0	3,329	95,02	34,92063492	4	47
P58876	HIST1H2BD	Histone H2B type 1-D OS	1,108	2,022	1,565	41,286	0,646	0,697	0,607	0,652	9,727	-0,617	4	4	47	0	0	3,329	95,02	34,92063492	4	47
Q93079	HIST1H2BH	Histone H2B type 1-H OS	1,108	2,022	1,565	41,286	0,646	0,697	0,607	0,652	9,727	-0,617	4	4	47	0	0	3,329	95,02	34,92063492	4	47
P06899	HIST1H2BJ	Histone H2B type 1-J OS	1,108	2,022	1,565	41,286	0,646	0,697	0,607	0,652	9,727	-0,617	3	3	46	0	0	2,511	95,02	27,77777778	3	46
O60814	HIST1H2BK	Histone H2B type 1-K OS	1,108	2,022	1,565	41,286	0,646	0,697	0,607	0,652	9,727	-0,617	4	4	47	0	0	3,329	95,02	34,92063492	4	47
Q99880	HIST1H2BL	Histone H2B type 1-L OS	1,108	2,022	1,565	41,286	0,646	0,697	0,607	0,652	9,727	-0,617	4	4	47	0	0	3,329	95,02	34,92063492	4	47
Q99879	HIST1H2BM	Histone H2B type 1-M OS	1,108	2,022	1,565	41,286	0,646	0,697	0,607	0,652	9,727	-0,617	4	4	47	0	0	3,329	95,02	34,92063492	4	47
Q99877	HIST1H2BN	Histone H2B type 1-N OS	1,108	2,022	1,565	41,286	0,646	0,697	0,607	0,652	9,727	-0,617	4	4	47	0	0	3,329	95,02	34,92063492	4	47
P23527	HIST1H2BO	Histone H2B type 1-O OS	1,108	2,022	1,565	41,286	0,646	0,697	0,607	0,652	9,727	-0,617	3	3	46	0	0	2,511	95,02	27,77777778	3	46
P62805	HIST1H4A	Histone H4 OS	1,181	1,684	1,433	24,829	0,519	1,091	0,642	0,866	36,620	-0,207	4	4	27	4	0	9	51,17	30,09708738	4	27
Q6FI13	HIST2H2AA3	Histone H2A type 2-A OS	0,045	0,622	0,333	122,471	-1,585	0,315	0,314	0,315	0,150	-1,668	3	7	73	0	6	70,969	162,08	57,69230769	7	73
Q16777	HIST2H2AC	Histone H2A type 2-C OS	0,045	0,622	0,333	122,471	-1,585	0,315	0,314	0,315	0,150	-1,668	3	7	73	0	0	145,78	162,08	58,13953488	7	73
Q16778	HIST2H2BE	Histone H2B type 2-E OS	1,108	2,022	1,565	41,286	0,646	0,697	0,607	0,652	9,727	-0,617	3	3	46	0	0	2,511	95,02	27,77777778	3	46
Q5QNW6	HIST2H2BF	Histone H2B type 2-F OS	1,108	2,022	1,565	41,286	0,646	0,697	0,607	0,652	9,727	-0,617	4	4	47	0	0	3,329	95,02	34,92063492	4	47
Q8N257	HIST3H2BB	Histone H2B type 3-B OS	1,108	2,022	1,565	41,286	0,646	0,697	0,607	0,652	9,727	-0,617	3	3	46	0	0	2,511	95,02	27,77777778	3	46
P09429	HMGB1	High mobility group protein B1 OS	0,427	0,469	0,448	6,629	-1,158	0,156	0,158	0,157	1,052	-2,673	12	14	90	7	4	80,113	130,51	52,55813953	14	90
B2RPK0	HMGB1P1	Putative high mobility group protein B1-like 1 OS	0,528	0,469	0,499	8,413	-1,004	0,217	0,228	0,222	3,392	-2,169	5	6	46	0	0	7,111	54,76	25,59241706	6	46
Q13151	HNRNPA0	Heterogeneous nuclear ribonucleoprotein A0 OS	1,339	1,718	1,528	17,520	0,612	0,873	0,610	0,742	25,017	-0,431	8	8	94	8	0	10,659	242,54	39,01639344	8	94
P09651	HNRNPA1	Heterogeneous nuclear ribonucleoprotein A1 OS	2,091	2,154	2,123	2,121	1,086	0,797	0,446	0,622	39,859	-0,686	17	19	287	6	0	315,228	850,02	44,08602151	19	287
Q32P51	HNRNPA1L2	Heterogeneous nuclear ribonucleoprotein A1-like 2 OS	2,088	2,167	2,128	2,626	1,089	0,800	0,464	0,632	37,546	-0,662	11	13	183	0	0	67,129	457,02	33,75	13	183
P22626	HNRNPA2B1	Heterogeneous nuclear ribonucleoproteins A2/B1 OS	1,333	2,295	1,814	37,492	0,859	1,254	0,662	0,958	43,696	-0,062	23	25	374	23	4	1127,838	988,57	66,00566572	25	374
P51991	HNRNPA3	Heterogeneous nuclear ribonucleoprotein A3 OS	1,548	2,617	2,083	36,298	1,058	0,932	0,589	0,761	31,878	-0,394	19	19	223	19	0	157,489	555,11	40,21164021	19	223
Q99729	HNRNPAB	Heterogeneous nuclear ribonucleoprotein A/B OS	1,171	1,182	1,177	0,681	0,235	0,997	0,870	0,934	9,670	-0,099	13	14	226	13	0	157,489	488,21	27,10843373	14	226
P07910	HNRNPC	Heterogeneous nuclear ribonucleoproteins C1/C2 OS	0,966	1,260	1,113	18,721	0,154	1,244	1,087	1,166	9,566	0,221	11	11	194	4	0	13,925	428,38	27,45098039	11	194
O60812	HNRNPCL1	Heterogeneous nuclear ribonucleoprotein C-like 1 OS	0,946	1,282	1,114	21,345	0,156	1,241	1,103	1,172	8,366	0,229	7	7	124	0	0	4,484	293,78	12,62798635	7	124
B2RXH8	HNRNPCL2	Heterogeneous nuclear ribonucleoprotein C-like 2 OS	0,946	1,282	1,114	21,345	0,156	1,241	1,103	1,172	8,366	0,229	7	7	124	0	0	4,484	293,78	12,62798635	7	124
P0DMR1	HNRNPCL4	Heterogeneous nuclear ribonucleoprotein C-like 4 OS	0,946	1,282	1,114	21,345	0,156	1,241	1,103	1,172	8,366	0,229	7	7	124	0	0	4,926	293,78	12,62798635	7	124
Q14103	HNRNPD	Heterogeneous nuclear ribonucleoprotein D0 OS	1,185	1,112	1,149	4,535	0,200	1,348	1,133	1,240	12,238	0,311	15	18	225	15	6	291,864	470,18	36,33802817	18	225
P52597	HNRNPF	Heterogeneous nuclear ribonucleoprotein F OS	20,000	4,549	12,275	89,010	3,618	1,057	0,923	0,990	9,568	-0,014	3	5	62	3	0	1,721	134,57	21,20481928	5	62
P31943	HNRNPH1	Heterogeneous nuclear ribonucleoprotein H OS	1,054	1,653	1,353	31,315	0,437	1,024	0,873	0,949	11,307	-0,076	8	15	178	8	12	19,893	403,37	52,78396437	15	178
P61978	HNRNPK	Heterogeneous nuclear ribonucleoprotein K	0,580	0,807	0,693	23,185	-0,528	1,692	1,734	1,713	1,748	0,776	37	37	1141	37	0	4174,319	2987,13	69,97840173	37	1141
P52272	HNRNPM	Heterogeneous nuclear ribonucleoprotein M OS	1,362	1,596	1,479	11,187	0,565	0,868	0,767	0,818	8,736	-0,291	14	14	137	14	0	1,848	320,39	25,89041096	14	137
O43390	HNRNPR	Heterogeneous nuclear ribonucleoprotein R OS	0,696	0,830	0,763	12,424	-0,391	1,380	1,349	1,364	1,607	0,448	29	37	816	29	0	324,233	1986,21	51,02685624	37	816
Q00839	HNRNPU	Heterogeneous nuclear ribonucleoprotein U OS	0,726	0,898	0,812	14,946	-0,300	1,271	1,210	1,240	3,459	0,311	36	36	407	36	0	43,101	926,11	44,36363636	36	407
Q9BUJ2	HNRNPUL1	Heterogeneous nuclear ribonucleoprotein U-like protein 1 OS	0,913	0,736	0,824	15,154	-0,279	1,055	1,382	1,219	18,937	0,285	17	17	116	17	0	2,754	179,58	25,58411215	17	116
P54652	HSPA2	Heat shock-related 70 kDa protein 2 OS	0,900	0,945	0,922	3,424	-0,117	1,137	1,040	1,089	6,301	0,122	7	10	177	0	0	2,44	504,83	14,39749609	10	177
P11021	HSPA5	78 kDa glucose-regulated protein OS	0,797	1,211	1,004	29,139	0,006	1,313	1,112	1,213	11,701	0,278	27	29	247	26	0	16,535	581,04	46,63608563	29	247
P11142	HSPA8	Heat shock cognate 71 kDa protein OS	0,884	1,051	0,968	12,201	-0,047	1,141	1,058	1,100	5,338	0,137	27	30	536	20	4	87,862	1394,64	54,64396285	30	536
P38646	HSPA9	Stress-70 protein, mitochondrial OS	0,947	1,187	1,067	15,905	0,094	1,314	1,128	1,221	10,810	0,288	9	9	77	9	0	1,434	140,12	20,91310751	9	77
P04792	HSPB1	Heat shock protein beta-1 OS	0,321	3,189	1,755	115,533	0,812	2,163	1,793	1,978	13,227	0,984	3	3	25	3	0	1,894	46,31	20	3	25
P10809	HSPD1	60 kDa heat shock protein, mitochondrial OS	0,208	1,531	0,869	107,662	-0,202	5,700	4,211	4,956	21,247	2,309	9	9	15	9	0	0,778	21,71	25,82897033	9	15
Q9Y6M1	IGF2BP2	Insulin-like growth factor 2 mRNA-binding protein 2 OS	1,478	1,541	1,510	2,966	0,594	0,636	0,524	0,580	13,647	-0,785	17	17	146	15	0	6,197	283,86	36,7278798	17	146
Q12905	ILF2	Interleukin enhancer-binding factor 2 OS	1,029	0,728	0,879	24,174	-0,187	1,026	0,960	0,993	4,698	-0,010	10	10	70	10	0	7,031	169,4	49,74358974	10	70
Q12906	ILF3	Interleukin enhancer-binding factor 3 OS	1,027	0,963	0,995	4,608	-0,007	0,950	0,837	0,894	8,943	-0,162	19	19	80	16	0	2,782	112,71	25,27964206	19	80
Q15046	KARS	Lysine--tRNA ligase OS	1,147	1,541	1,344	20,709	0,427	0,784	0,456	0,620	37,318	-0,690	11	11	48	11	0	2,06	71,57	21,94304858	11	48
Q86UP2	KTN1	Kinectin OS	0,837	0,819	0,828	1,552	-0,273	0,879	0,356	0,617	59,922	-0,696	13	13	66	13	0	0,704	93,06	15,62269713	13	66
P42704	LRPPRC	Leucine-rich PPR motif-containing protein, mitochondrial	3,472	2,487	2,980	23,392	1,575	0,342	0,280	0,311	14,112	-1,687	44	44	225	44	0	3,748	425,14	43,1133429	44	225
Q96AG4	LRRC59	Leucine-rich repeat-containing protein 59 OS	0,839	1,112	0,976	19,789	-0,036	1,236	0,779	1,008	32,064	0,011	12	12	157	12	0	45,416	452,13	39,41368078	12	157
Q9Y333	LSM2	U6 snRNA-associated Sm-like protein LSm2	0,779	0,553	0,666	23,966	-0,587	1,284	0,934	1,109	22,274	0,149	3	3	22	3	0	4,179	32,45	48,42105263	3	22
Q96GA3	LTV1	Protein LTV1 homolog OS	0,824	1,188	1,006	25,556	0,008	1,021	0,943	0,982	5,593	-0,026	6	6	37	5	0	0,931	37,9	14,10526316	6	37
Q9NX58	LYAR	Cell growth-regulating nucleolar protein OS	1,026	1,036	1,031	0,709	0,044	1,179	1,195	1,187	0,953	0,248	8	8	66	8	0	4,179	171,45	20,31662269	8	66
P43243	MATR3	Matrin-3	0,128	0,188	0,158	26,731	-2,664	7,541	7,786	7,664	2,264	2,938	42	42	366	42	0	48,417	848,8	58,32349469	42	366
P41218	MNDA	Myeloid cell nuclear differentiation antigen OS	1,015	0,898	0,957	8,699	-0,064	0,908	0,877	0,892	2,483	-0,164	17	17	134	17	0	10,721	264,73	56,26535627	17	134
Q13724	MOGS	Mannosyl-oligosaccharide glucosidase OS	0,997	1,118	1,058	8,135	0,081	1,011	0,711	0,861	24,615	-0,216	18	18	133	18	0	4,142	272,5	35,24492234	18	133
P26038	MSN	Moesin OS	1,424	2,282	1,853	32,767	0,890	1,584	1,450	1,517	6,262	0,601	9	9	42	6	0	1,075	38,8	17,50433276	9	42
Q86UE4	MTDH	Protein LYRIC OS	1,277	1,644	1,461	17,764	0,547	0,797	0,579	0,688	22,405	-0,540	20	20	215	20	0	26,123	464,56	50,68728522	20	215
Q14764	MVP	Major vault protein	0,273	0,245	0,259	7,635	-1,947	2,850	2,407	2,629	11,908	1,394	52	52	536	52	0	99	1331,49	73,01231803	52	536
P55209	NAP1L1	Nucleosome assembly protein 1-like 1 OS	0,940	1,625	1,283	37,758	0,359	0,897	0,945	0,921	3,660	-0,119	6	6	59	5	0	4,412	144,77	17,64705882	6	59
P19338	NCL	Nucleolin	0,852	0,758	0,805	8,260	-0,314	1,079	1,081	1,080	0,109	0,111	51	51	1395	51	0	1048,14	3759,13	43,94366197	51	1395
O00567	NOP56	Nucleolar protein 56 OS	0,973	1,179	1,076	13,538	0,106	1,019	0,918	0,968	7,377	-0,047	16	16	84	16	0	3,642	181,32	37,03703704	16	84
Q9Y2X3	NOP58	Nucleolar protein 58 OS	0,830	1,035	0,932	15,522	-0,101	0,969	0,848	0,908	9,421	-0,139	11	11	72	11	0	3,299	136,55	28,92249527	11	72
P06748	NPM1	Nucleophosmin OS	0,744	0,981	0,863	19,485	-0,213	0,897	0,809	0,853	7,350	-0,229	10	10	68	10	0	9	82,43	44,55782313	10	68
P11940	PABPC1	Polyadenylate-binding protein 1 OS	1,107	0,904	1,006	14,229	0,008	0,820	0,849	0,835	2,456	-0,260	14	25	273	5	0	17,836	549,75	44,02515723	25	273
Q4VXU2	PABPC1L	Polyadenylate-binding protein 1-like OS	1,120	0,718	0,919	30,880	-0,122	0,793	0,813	0,803	1,702	-0,317	3	4	38	0	0	0,512	58,19	7,166123779	4	38
Q9H361	PABPC3	Polyadenylate-binding protein 3 OS	1,114	0,909	1,011	14,310	0,016	0,827	0,847	0,837	1,689	-0,256	7	9	95	0	0	1,565	162,91	16,48177496	9	95
Q13310	PABPC4	Polyadenylate-binding protein 4 OS	1,109	1,192	1,151	5,101	0,202	0,862	0,797	0,830	5,539	-0,269	15	26	299	13	18	20,135	649,01	50,46583851	26	299
P09874	PARP1	Poly [ADP-ribose] polymerase 1 OS	0,679	0,659	0,669	2,115	-0,581	2,071	1,093	1,582	43,684	0,662	24	24	200	24	0	3,994	293,35	33,33333333	24	200
Q15365	PCBP1	Poly(rC)-binding protein 1 OS	0,706	0,852	0,779	13,219	-0,360	1,460	1,429	1,445	1,517	0,531	11	18	266	11	0	110,588	575,78	79,49438202	18	266
Q15366	PCBP2	Poly(rC)-binding protein 2 OS	0,653	0,696	0,675	4,472	-0,568	1,610	1,635	1,623	1,060	0,698	11	19	382	11	13	280,838	912,98	83,83561644	19	382
O43660	PLRG1	Pleiotropic regulator 1 OS	1,030	1,397	1,213	21,360	0,279	0,879	0,868	0,874	0,836	-0,195	6	6	43	6	0	1,929	59,32	25,09727626	6	43
Q6S8J3	POTEE	POTE ankyrin domain family member E OS	0,311	2,153	1,232	105,722	0,301	4,699	0,798	2,749	100,344	1,459	4	8	130	0	0	1,13	301,54	8,093023256	8	130
P62937	PPIA	Peptidyl-prolyl cis-trans isomerase A	0,286	21,356	10,821	137,684	3,436	5,075	10,444	7,760	48,927	2,956	6	6	29	5	0	3,394	24,25	60,60606061	6	29
O43447	PPIH	Peptidyl-prolyl cis-trans isomerase H OS	1,028	1,102	1,065	4,913	0,091	0,929	0,852	0,891	6,114	-0,167	9	9	82	9	0	18,307	187,33	61,5819209	9	82
Q9UMS4	PRPF19	Pre-mRNA-processing factor 19 OS	1,147	1,265	1,206	6,939	0,270	0,978	0,921	0,950	4,219	-0,075	11	11	58	11	0	8,047	79,01	46,03174603	11	58
O43395	PRPF3	U4/U6 small nuclear ribonucleoprotein Prp3 OS	1,049	1,659	1,354	31,878	0,437	1,014	0,891	0,952	9,157	-0,070	8	8	53	8	0	1,424	82,29	17,27672035	8	53
O43172	PRPF4	U4/U6 small nuclear ribonucleoprotein Prp4 OS	1,038	0,639	0,839	33,669	-0,254	0,937	0,988	0,963	3,698	-0,055	14	14	70	14	0	3,299	125,55	36,39846743	14	70
O75475	PSIP1	PC4 and SFRS1-interacting protein OS	1,103	0,640	0,872	37,566	-0,198	0,618	0,598	0,608	2,248	-0,718	5	6	40	5	0	0,983	48,04	15,28301887	6	40
P26599	PTBP1	Polypyrimidine tract-binding protein 1 OS	0,147	0,168	0,158	9,129	-2,667	8,827	9,071	8,949	1,925	3,162	35	39	840	35	6	8316,638	2454,78	77,77777778	39	840
O95758	PTBP3	Polypyrimidine tract-binding protein 3 OS	0,078	0,271	0,174	78,117	-2,521	11,439	9,959	10,699	9,779	3,419	9	13	93	9	0	5,062	199,96	31,70289855	13	93
Q13610	PWP1	Periodic tryptophan protein 1 homolog OS	0,857	0,813	0,835	3,754	-0,260	0,981	0,917	0,949	4,769	-0,076	11	11	55	11	0	3,642	45,02	31,73652695	11	55
Q9UKM9	RALY	RNA-binding protein Raly OS	0,849	0,724	0,787	11,266	-0,346	1,220	1,148	1,184	4,281	0,243	12	12	125	11	0	14,849	299,42	42,48366013	12	125
Q9BTD8	RBM42	RNA-binding protein 42 OS	0,247	0,525	0,386	51,009	-1,374	3,862	3,969	3,915	1,938	1,969	10	10	158	10	0	26,826	348,99	28,33333333	10	158
P38159	RBMX	RNA-binding motif protein, X chromosome OS	0,438	0,489	0,464	7,882	-1,109	2,396	2,156	2,276	7,457	1,186	8	17	247	6	12	10,498	623,33	30,17902813	17	247
P35241	RDX	Radixin OS	1,406	2,456	1,931	38,459	0,949	1,871	1,462	1,667	17,351	0,737	3	3	29	0	0	0,334	19,95	4,459691252	3	29
P15927	RPA2	Replication protein A 32 kDa subunit OS	3,500	5,045	4,273	25,575	2,095	0,242	0,167	0,204	25,928	-2,293	8	8	25	8	0	4,179	38,95	64,44444444	8	25
P35244	RPA3	Replication protein A 14 kDa subunit OS	8,324	2,934	5,629	67,711	2,493	0,318	0,277	0,298	9,745	-1,749	6	6	30	6	0	16,783	85	70,24793388	6	30
P27635	RPL10	60S ribosomal protein L10 OS	1,292	1,501	1,397	10,598	0,482	0,817	0,856	0,837	3,295	-0,257	7	7	58	5	0	16,013	124,37	35,04672897	7	58
P62906	RPL10A	60S ribosomal protein L10a OS	1,324	1,134	1,229	10,980	0,297	0,688	0,643	0,665	4,784	-0,588	7	7	114	7	0	17,478	246,93	35,02304147	7	114
P62913	RPL11	60S ribosomal protein L11 OS	0,963	1,098	1,031	9,260	0,044	1,017	0,994	1,006	1,641	0,008	5	5	50	5	0	11,915	120,74	38,76404494	5	50
P30050	RPL12	60S ribosomal protein L12 OS	1,136	1,175	1,155	2,367	0,208	0,797	0,765	0,781	2,927	-0,356	5	5	76	5	0	11,328	190,65	44,84848485	5	76
P26373	RPL13	60S ribosomal protein L13 OS	1,108	0,549	0,828	47,747	-0,272	0,745	0,805	0,775	5,477	-0,368	7	7	55	7	0	9	61,78	28,90995261	7	55
P50914	RPL14	60S ribosomal protein L14 OS	1,215	1,581	1,398	18,493	0,484	0,728	0,669	0,699	5,973	-0,518	4	4	68	4	0	4,623	160,28	20,93023256	4	68
P61313	RPL15	60S ribosomal protein L15 OS	1,321	1,091	1,206	13,535	0,270	0,696	0,749	0,722	5,221	-0,469	7	7	31	7	0	4,179	48,54	31,8627451	7	31
Q07020	RPL18	60S ribosomal protein L18 OS	1,235	1,222	1,228	0,729	0,297	0,717	0,646	0,682	7,330	-0,553	5	5	63	5	0	11,915	161,82	31,38297872	5	63
P84098	RPL19	60S ribosomal protein L19 OS	1,084	1,105	1,094	1,325	0,130	0,855	0,828	0,842	2,296	-0,249	5	5	28	5	0	6,743	51,78	17,85714286	5	28
P46778	RPL21	60S ribosomal protein L21 OS	1,173	0,205	0,689	99,354	-0,537	0,817	0,667	0,742	14,323	-0,430	7	7	35	6	0	11,915	60,66	46,875	7	35
P35268	RPL22	60S ribosomal protein L22 OS	1,000	0,899	0,950	7,545	-0,075	1,033	1,035	1,034	0,182	0,048	10	10	372	9	0	999999	926,08	69,53125	10	372
P62829	RPL23	60S ribosomal protein L23 OS	0,929	0,678	0,803	22,123	-0,316	1,021	1,042	1,031	1,440	0,044	10	10	129	10	0	78,433	282,16	55	10	129
P62750	RPL23A	60S ribosomal protein L23a OS	0,890	0,871	0,881	1,552	-0,183	0,911	1,311	1,111	25,441	0,152	8	8	57	8	0	45,416	105,93	51,28205128	8	57
P83731	RPL24	60S ribosomal protein L24 OS	0,867	2,255	1,561	62,887	0,642	0,996	0,939	0,967	4,191	-0,048	5	5	74	5	0	11,589	157,61	27,38853503	5	74
P61353	RPL27	60S ribosomal protein L27 OS	1,000	1,621	1,310	33,530	0,390	0,629	0,626	0,627	0,301	-0,673	8	8	85	8	0	680,292	100,72	54,41176471	8	85
P62888	RPL30	60S ribosomal protein L30 OS	1,319	1,508	1,414	9,421	0,499	0,811	0,798	0,805	1,113	-0,314	6	6	84	6	0	41,17	232,96	58,26086957	6	84
P62899	RPL31	60S ribosomal protein L31 OS	0,848	1,340	1,094	31,840	0,129	0,723	0,788	0,756	6,084	-0,404	3	3	34	3	0	6,197	54,51	25,6	3	34
P62910	RPL32	60S ribosomal protein L32 OS	1,305	0,882	1,094	27,353	0,129	0,717	0,778	0,748	5,738	-0,420	4	4	43	4	0	30,623	72,73	29,62962963	4	43
P42766	RPL35	60S ribosomal protein L35 OS	0,952	1,099	1,025	10,161	0,036	0,889	1,089	0,989	14,347	-0,016	6	6	94	6	0	680,292	213,96	30,89430894	6	94
P63173	RPL38	60S ribosomal protein L38 OS	0,907	1,264	1,085	23,263	0,118	0,966	0,992	0,979	1,926	-0,031	7	7	130	7	0	999999	352,05	57,14285714	7	130
P36578	RPL4	60S ribosomal protein L4 OS	1,053	1,176	1,114	7,806	0,156	0,761	0,749	0,755	1,155	-0,405	15	15	204	15	0	10,659	460,25	36,06557377	15	204
P46777	RPL5	60S ribosomal protein L5 OS	1,597	1,323	1,460	13,270	0,546	0,550	0,554	0,552	0,598	-0,857	11	11	115	11	0	20,544	218,32	40,4040404	11	115
Q02878	RPL6	60S ribosomal protein L6 OS	1,317	1,526	1,422	10,412	0,508	0,723	0,675	0,699	4,858	-0,517	14	14	99	14	0	10,288	135,12	47,22222222	14	99
P18124	RPL7	60S ribosomal protein L7 OS	1,413	1,430	1,422	0,845	0,508	0,634	0,548	0,591	10,295	-0,760	18	18	123	18	0	99	213,36	54,43548387	18	123
P62424	RPL7A	60S ribosomal protein L7a OS	1,281	2,066	1,674	33,151	0,743	0,564	0,572	0,568	1,037	-0,816	16	16	128	16	0	45,416	270,61	46,61654135	16	128
P32969	RPL9	60S ribosomal protein L9 OS	0,601	0,690	0,645	9,754	-0,632	1,778	1,563	1,671	9,086	0,740	13	13	175	13	0	810,131	432,33	76,04166667	13	175
P05388	RPLP0	60S acidic ribosomal protein P0 OS	1,007	1,375	1,191	21,848	0,252	0,795	0,790	0,792	0,476	-0,336	14	14	112	5	0	22,357	216,5	52,68138801	14	112
Q8NHW5	RPLP0P6	60S acidic ribosomal protein P0-like OS	1,048	1,375	1,211	19,108	0,277	0,846	0,780	0,813	5,740	-0,299	9	9	93	0	0	6,943	183,25	33,4384858	9	93
P05387	RPLP2	60S acidic ribosomal protein P2 OS	0,998	1,845	1,422	42,145	0,508	0,958	0,818	0,888	11,177	-0,172	9	9	128	9	0	3161,278	384,34	80,86956522	9	128
P46783	RPS10	40S ribosomal protein S10 OS	0,910	1,096	1,003	13,160	0,004	1,251	0,910	1,081	22,309	0,112	11	11	231	7	0	141,51	536,9	58,78787879	11	231
Q9NQ39	RPS10P5	Putative 40S ribosomal protein S10-like OS	1,065	1,117	1,091	3,370	0,126	1,297	0,886	1,091	26,651	0,126	4	4	81	0	0	7,254	160,26	17,61363636	4	81
P25398	RPS12	40S ribosomal protein S12 OS	0,920	0,904	0,912	1,241	-0,133	0,891	0,873	0,882	1,416	-0,181	11	11	286	11	0	37274,94	775,2	65,15151515	11	286
P62263	RPS14	40S ribosomal protein S14 OS	0,835	0,912	0,874	6,259	-0,195	1,065	1,070	1,068	0,353	0,094	12	12	144	12	0	5178,475	387,14	70,1986755	12	144
P62244	RPS15A	40S ribosomal protein S15a OS	0,945	1,076	1,011	9,167	0,015	1,108	1,102	1,105	0,405	0,144	10	10	119	10	0	52,367	245,87	61,53846154	10	119
P62249	RPS16	40S ribosomal protein S16 OS	0,909	1,011	0,960	7,513	-0,059	1,072	1,136	1,104	4,100	0,142	9	9	77	9	0	13,678	137,16	50	9	77
P08708	RPS17	40S ribosomal protein S17 OS	0,929	1,191	1,060	17,453	0,084	0,930	1,263	1,097	21,431	0,133	7	7	122	7	0	14676,99	267,9	52,59259259	7	122
P62269	RPS18	40S ribosomal protein S18 OS	0,724	0,882	0,803	13,972	-0,317	1,253	1,183	1,218	4,084	0,284	10	10	89	10	0	20,544	163,63	46,71052632	10	89
P39019	RPS19	40S ribosomal protein S19 OS	0,519	0,744	0,631	25,207	-0,664	2,127	1,901	2,014	7,946	1,010	12	12	252	12	0	463,159	562,55	58,62068966	12	252
P15880	RPS2	40S ribosomal protein S2 OS	1,228	2,239	1,734	41,212	0,794	0,796	0,708	0,752	8,241	-0,411	8	8	83	8	0	3,329	174,51	31,05802048	8	83
P60866	RPS20	40S ribosomal protein S20 OS	0,941	0,562	0,751	35,697	-0,413	0,980	0,927	0,953	3,932	-0,069	5	5	67	5	0	99	136,87	29,41176471	5	67
P62266	RPS23	40S ribosomal protein S23 OS	1,013	0,707	0,860	25,182	-0,217	1,120	0,989	1,054	8,763	0,076	6	6	57	6	0	12,335	108,9	43,35664336	6	57
P62847	RPS24	40S ribosomal protein S24 OS	0,961	1,422	1,191	27,343	0,253	0,905	1,068	0,987	11,705	-0,019	5	5	72	5	0	25,827	156,11	30,07518797	5	72
P62851	RPS25	40S ribosomal protein S25 OS	0,911	0,919	0,915	0,644	-0,128	1,047	1,075	1,061	1,910	0,085	7	7	98	7	0	145,78	235,23	32,8	7	98
P62854	RPS26	40S ribosomal protein S26 OS	1,111	0,946	1,029	11,340	0,041	0,858	0,763	0,810	8,292	-0,304	4	4	36	1	0	9	59,88	44,34782609	4	36
P62857	RPS28	40S ribosomal protein S28 OS	0,739	0,859	0,799	10,647	-0,323	1,025	1,063	1,044	2,597	0,062	7	7	111	7	0	46414,89	292,7	60,86956522	7	111
P23396	RPS3	40S ribosomal protein S3 OS	0,887	1,224	1,056	22,621	0,078	0,968	1,032	1,000	4,506	-0,022	15	15	178	15	0	28,764	325,33	62,55144033	15	178
P61247	RPS3A	40S ribosomal protein S3a OS	0,998	1,623	1,311	33,737	0,390	0,932	0,972	0,952	2,956	-0,071	19	19	174	17	0	67,587	313,35	64,5454545	19	174
P62701	RPS4X	40S ribosomal protein S4, X isoform OS	0,767	0,679	0,723	8,541	-0,468	1,215	1,262	1,239	2,645	0,309	17	20	186	15	3	62,096	262,97	63,878327	20	186
P46782	RPS5	40S ribosomal protein S5 OS	0,825	0,937	0,881	8,993	-0,183	0,935	0,937	0,936	0,155	-0,095	12	12	197	12	0	999	594,79	62,74509804	12	197
P62753	RPS6	40S ribosomal protein S6 OS	0,846	0,766	0,806	7,016	-0,311	1,071	1,094	1,083	1,524	0,115	9	9	101	9	0	13,251	290,72	28,1124498	9	101
P62081	RPS7	40S ribosomal protein S7 OS	1,447	1,342	1,394	5,554	0,480	1,260	1,009	1,134	15,655	0,182	15	15	160	15	0	656,933	277,69	59,79381443	15	160
P62241	RPS8	40S ribosomal protein S8 OS	1,053	1,102	1,078	3,237	0,108	0,747	0,744	0,745	0,285	-0,424	12	12	170	12	0	78,433	222,83	59,61538462	12	170
P46781	RPS9	40S ribosomal protein S9 OS	1,359	1,529	1,444	8,342	0,530	0,646	0,617	0,632	3,298	-0,663	2	2	4	2	0	2,162	11,04	22,68041237	2	4
P08865	RPSA	40S ribosomal protein SA OS	0,697	1,493	1,095	51,401	0,130	1,109	1,162	1,136	3,278	0,184	17	17	174	17	0	17,478	[illegible]	[illegible]	17	174
Q9Y3I0	RTCB	tRNA-splicing ligase RtcB homolog OS	0,415	0,423	0,419	1,435	-1,256	3,253	2,360	2,807	22,490	1,489	10	10	39	10	0	1,712	72,62	29,7029703	10	39
Q15393	SF3B3	Splicing factor 3B subunit 3 OS	0,652	1,146	0,899	38,859	-0,154	1,430	1,474	1,452	2,175	0,538	12	12	53	12	0	0,892	74,93	14,7082991	12	53
Q9GZT3	SLIRP	SRA stem-loop-interacting RNA-binding protein, mitochondrial OS	2,971	2,699	2,835	6,801	1,503	0,344	0,290	0,317	11,897	-1,657	6	6	[illegible]	6	0	[illegible]	[illegible]	[illegible]	6	[illegible]
P55769	SNU13	NHP2-like protein 1 OS	0,881	1,063	0,972	13,192	-0,041	0,994	0,870	0,932	9,408	-0,102	6	6	[illegible]	6	0	[illegible]	[illegible]	[illegible]	6	[illegible]
Q5T280	SPOUT1	Putative methyltransferase C9orf114 OS	1,149	1,737	1,443	28,794	0,529	0,858	0,858	0,858	0,055	-0,221	7	7	37	7	0	2,548	75,75	25	7	37
P37108	SRP14	Signal recognition particle 14 kDa protein OS	1,108	0,932	1,020	12,201	0,029	0,903	0,844	0,874	4,772	-0,195	12	12	197	12	0	2370,374	454,37	76,47058824	12	197
P49458	SRP9	Signal recognition particle 9 kDa protein OS	[illegible]	[illegible]	[illegible]	[illegible]	[illegible]	[illegible]	[illegible]	[illegible]	[illegible]	[illegible]	[illegible]	[illegible]	[illegible]	[illegible]	[illegible]	[illegible]	[illegible]	[illegible]	[illegible]	[illegible]
O75494	SRSF10	Serine/arginine-rich splicing factor 10 OS	6,804	11,091	8,947	33,883	3,161	0,283	0,204	0,244	22,829	-2,037	5	5	35	4	0	4,179	90,38	27,8641214	5	35
P84103	SRSF3	Serine/arginine-rich splicing factor 3 OS	[illegible]	[illegible]	[illegible]	[illegible]	[illegible]	[illegible]	[illegible]	[illegible]	[illegible]	[illegible]	[illegible]	[illegible]	[illegible]	[illegible]	[illegible]	[illegible]	[illegible]	[illegible]	[illegible]	[illegible]
Q16629	SRSF7	Serine/arginine-rich splicing factor 7 OS	[illegible]	[illegible]	[illegible]	[illegible]	[illegible]	[illegible]	[illegible]	[illegible]	[illegible]	[illegible]	[illegible]	[illegible]	[illegible]	[illegible]	[illegible]	[illegible]	[illegible]	[illegible]	[illegible]	[illegible]
P05455	SSB	Lupus La protein OS	[illegible]	[illegible]	[illegible]	[illegible]	[illegible]	[illegible]	[illegible]	[illegible]	[illegible]	[illegible]	[illegible]	[illegible]	[illegible]	[illegible]	[illegible]	[illegible]	[illegible]	[illegible]	[illegible]	[illegible]
Q08945	SSRP1	FACT complex subunit SSRP1 OS	[illegible]	[illegible]	[illegible]	[illegible]	[illegible]	[illegible]	[illegible]	[illegible]	[illegible]	[illegible]	[illegible]	[illegible]	[illegible]	[illegible]	[illegible]	[illegible]	[illegible]	[illegible]	[illegible]	[illegible]
O60506	SYNCRIP	Heterogeneous nuclear ribonucleoprotein Q	[illegible]	[illegible]	[illegible]	[illegible]	[illegible]	[illegible]	[illegible]	[illegible]	[illegible]	[illegible]	44	52	1594	44	16	23712,74	4718,12	60,03210273	52	1594
Q00059	TFAM	Transcription factor A, mitochondrial OS	[illegible]	[illegible]	[illegible]	[illegible]	[illegible]	[illegible]	[illegible]	[illegible]	[illegible]	[illegible]	[illegible]	[illegible]	[illegible]	[illegible]	[illegible]	[illegible]	[illegible]	[illegible]	[illegible]	[illegible]
Q71U36	TUBA1A	Tubulin alpha-1A chain OS	0,592	2,820	1,706	92,319	0,771	1,393	1,731	1,562	15,284	0,644	[illegible]	[illegible]	[illegible]	[illegible]	[illegible]	[illegible]	[illegible]	[illegible]	[illegible]	[illegible]
P68363	TUBA1B	Tubulin alpha-1B chain OS	[illegible]	[illegible]	[illegible]	[illegible]	[illegible]	[illegible]	[illegible]	[illegible]	[illegible]	[illegible]	[illegible]	[illegible]	[illegible]	[illegible]	[illegible]	[illegible]	[illegible]	[illegible]	[illegible]	[illegible]
Q9BQE3	TUBA1C	Tubulin alpha-1C chain OS	[illegible]	[illegible]	[illegible]	[illegible]	[illegible]	[illegible]	[illegible]	[illegible]	[illegible]	[illegible]	[illegible]	[illegible]	[illegible]	[illegible]	[illegible]	[illegible]	[illegible]	[illegible]	[illegible]	[illegible]
P68366	TUBA4A	Tubulin alpha-4A chain OS	[illegible]	[illegible]	[illegible]	[illegible]	[illegible]	[illegible]	[illegible]	[illegible]	[illegible]	[illegible]	[illegible]	[illegible]	[illegible]	[illegible]	[illegible]	[illegible]	[illegible]	[illegible]	[illegible]	[illegible]
P07437	TUBB	Tubulin beta chain OS	[illegible]	[illegible]	[illegible]	[illegible]	[illegible]	[illegible]	[illegible]	[illegible]	[illegible]	[illegible]	[illegible]	[illegible]	[illegible]	[illegible]	[illegible]	[illegible]	[illegible]	[illegible]	[illegible]	[illegible]
P04350	TUBB4A	Tubulin beta-4A chain OS	[illegible]	[illegible]	[illegible]	[illegible]	[illegible]	[illegible]	[illegible]	[illegible]	[illegible]	[illegible]	[illegible]	[illegible]	[illegible]	[illegible]	[illegible]	[illegible]	[illegible]	[illegible]	[illegible]	[illegible]
P68371	TUBB4B	Tubulin beta-4B chain	[illegible]	[illegible]	[illegible]	[illegible]	[illegible]	[illegible]	[illegible]	[illegible]	[illegible]	[illegible]	[illegible]	[illegible]	[illegible]	[illegible]	[illegible]	[illegible]	[illegible]	[illegible]	[illegible]	[illegible]
Q01081	U2AF1	Splicing factor U2AF 35 kDa subunit OS	[illegible]	[illegible]	[illegible]	[illegible]	[illegible]	[illegible]	[illegible]	[illegible]	[illegible]	[illegible]	[illegible]	[illegible]	[illegible]	[illegible]	[illegible]	[illegible]	[illegible]	[illegible]	[illegible]	[illegible]
P26368	U2AF2	Splicing factor U2AF 65 kDa subunit OS	[illegible]	[illegible]	[illegible]	[illegible]	[illegible]	[illegible]	[illegible]	[illegible]	[illegible]	[illegible]	[illegible]	[illegible]	[illegible]	[illegible]	[illegible]	[illegible]	[illegible]	[illegible]	[illegible]	[illegible]
Q9H0D6	XRN2	5'-3' exoribonuclease 2 OS	[illegible]	[illegible]	[illegible]	[illegible]	[illegible]	[illegible]	[illegible]	[illegible]	[illegible]	[illegible]	[illegible]	[illegible]	[illegible]	[illegible]	[illegible]	[illegible]	[illegible]	[illegible]	[illegible]	[illegible]
P67809	YBX1	Nuclease-sensitive element-binding protein 1 OS	[illegible]	[illegible]	[illegible]	[illegible]	[illegible]	[illegible]	[illegible]	[illegible]	[illegible]	[illegible]	6	12	142	6	11	116,877	234,07	48,7654321	12	142

Supplementary Table: siRNAs used in the study

No.	Gene	Assay ID	Library	Sense siRNA Sequence	Antisense siRNA sequence
87	SRSF1	s12725	Ambion Silencer Select	GACCUAUGCAGUUCGAAAAtt	UUUUCGAACUGCAUAGGUCat
88	SRSF1	s12726	Ambion Silencer Select	GGAUAACACUAAGUUUAGAtt	UCUAAACUUAGUGUUAUCCag
89	SRSF1	s12727	Ambion Silencer Select	GCAUCUACGUGGGUAACUUtt	AAGUUACCCACGUAGAUGCgg
90	SRSF2	J-019711-05 (Dharmacon)	Dharmacon ON-TARGETplus	Sequence not provided by vendor	Sequence not provided by vendor
91	SRSF2	J-019711-06 (Dharmacon)	Dharmacon ON-TARGETplus	Sequence not provided by vendor	Sequence not provided by vendor
92	SRSF2	J-019711-08 (Dharmacon)	Dharmacon ON-TARGETplus	Sequence not provided by vendor	Sequence not provided by vendor
93	SRSF3	s12731	Ambion Silencer Select	CGCUGUCUCGGGAGAGAAAtt	UUUCUCUCCCGAGACAGCGat
94	SRSF3	s12732	Ambion Silencer Select	AGAGCUAGAUGGAAGAACAtt	UGUUCUUCCAUCUAGCUCUcg
95	SRSF3	s12733	Ambion Silencer Select	GCAACAAGACGGAAUUGGAtt	UCCAAUUCCGUCUUGUUGCca
96	SRSF4	s12734	Ambion Silencer Select	CGCACAGAGUACAGACUUAtt	UAAGUCUGUACUCUGUGCGag
97	SRSF4	s12735	Ambion Silencer Select	CCUUUGUGGUGAGCGAGUAtt	UACUCGCUCACCACAAAGGtc
98	SRSF4	s12736	Ambion Silencer Select	CAGUCAUUCUAAGAGUAGAtt	UCUACUCUUAGAAUGACUGct
99	SRSF5	s12737	Ambion Silencer Select	CCACCUGUAAGAACAGAAAtt	UUUCUGUUCUUACAGGUGGag
100	SRSF5	s12738	Ambion Silencer Select	GGACGAUACUCUGACCGUUtt	AACGGUCAGAGUAUCGUCCtc
101	SRSF5	SRSF5	Ambion Silencer Select	CCUCUUAUGGUGACUUAAAtt	UUUAAGUCACCAUAAGAGGca
102	SRSF6	s12740	Ambion Silencer Select	CAAAUGAGGGUGUAAUUGAtt	UCAAUUACACCCUCAUUUGtt
103	SRSF6	s12741	Ambion Silencer Select	CGUUCUCGAUCAAAAGGCAtt	UGCCUUUUGAUCGAGAACGtg
104	SRSF6	s12742	Ambion Silencer Select	GGGUGAUAUAAAGUCAAAAtt	UUUUGACUUUAUAUCACCCtt
105	SRSF7	s12743	Ambion Silencer Select	CUCUCUUCGUAGAUCAAGAtt	UCUUGAUCUACGAAGAGAGat
106	SRSF7	s12744	Ambion Silencer Select	CGAAGAUCCUAGAGAUGCAtt	UGCAUCUCUAGGAUCUUCGaa
107	SRSF7	s12745	Ambion Silencer Select	GGCUUUCAGUUAUUAUGGUtt	ACCAUAAUAACUGAAAGCCct
108	SRSF8	s21495	Ambion Silencer Select	GCUCAUCUGGUUACAGUAAtt	UUACUGUAACCAGAUGAGCtg
109	SRSF8	s21496	Ambion Silencer Select	GCAGAUCUCGCUACAGGGAtt	UCCCUGUAGCGAGAUCUGCtg
110	SRSF8	s21497	Ambion Silencer Select	GCAUCAACCUCCAAAUCGAtt	UCGAUUUGGAGGUUGAUGCag
111	SRSF9	s16546	Ambion Silencer Select	GGUCGAGUAUCUCAGAAAAtt	UUUUCUGAGAUACUCGACCat
112	SRSF9	s16547	Ambion Silencer Select	CCAAAUUCCGCUCUCAUGAtt	UCAUGAGAGCGGAAUUUGGtg
113	SRSF9	s16548	Ambion Silencer Select	ACAUCCGAGUUUAUCCUGAtt	UCAGGAUAAACUCGGAUGUag
114	SRSF10	s21156	Ambion Silencer Select	GGAUGUUCGUGAUGCUGAAtt	UUCAGCAUCACGAACAUCCtc
115	SRSF10	s21157	Ambion Silencer Select	GUCUUUUGAUUACAACUAUtt	AUAGUUGUAAUCAAAAGACcg
116	SRSF10	s21158	Ambion Silencer Select	UGGUCGUUAUGGUCCUAUAtt	UAUAGGACCAUAACGACCAaa
117	SRSF11	s17769	Ambion Silencer Select	CUUUGAUAGUCGUACCAUAtt	UAUGGUACGACUAUCAAAGct
118	SRSF11	s17770	Ambion Silencer Select	CUAUGAAAAGAGUACGAGAtt	UCUCGUACUCUUUUCAUAGct
119	SRSF12	s43932	Ambion Silencer Select	GUCUAAAUCUCGUUCCAAAtt	UUUGGAACGAGAUUUAGACtt
120	SRSF12	s43933	Ambion Silencer Select	GCCUUAAAGAGUCUCGACAtt	UGUCGAGACUCUUUAAGGCtg
121	SRSF12	s43934	Ambion Silencer Select	GGAAGACAUUCUGACUCAAtt	UUGAGUCAGAAUGUCUUCCat
122	HNRNPA0	s21543	Ambion Silencer Select	CGGUCGCAGUAAUAGUGGAtt	UCCACUAUUACUGCGACCGcc
123	hnRNPA1	s223858	Ambion Silencer Select	AGUUCACCAUUAAAAGGGATT	UCCCUUUUAAUGGUGAACUTC
124	hnRNPA1	s223859	Ambion Silencer Select	AUUGAAUAAUGGUACCAGATT	UCUGGUACCAUUAUUCAAUTT
125	hnRNPA1	s223860	Ambion Silencer Select	GAAUGGUUAUAAAAGUGAUtt	AUCACUUUUAUAACCAUUCca
126	hnRNPA1L2	s195754	Ambion Silencer Select	GGCUAUGGCGUUUCCAGCAtt	UGCUGGAAACGCCAUAGCCac
127	hnRNPA1L2	s195755	Ambion Silencer Select	UUGUGUGGCGUUGUAUUCAta	UUGUGUGGCGUUGUAUUCA
128	hnRNPA1L2	s195756	Ambion Silencer Select	CUAUGAAUACAACGCCACATT	UGUGGCGUUGUAUUCAUAGCT
129	hnRNPA2B1	s6713	Ambion Silencer Select	GCAACCUUCUAACUACGGUtt	ACCGUAGUUAGAAGGUUGCtg
130	hnRNPA2B1	s6714	Ambion Silencer Select	CAUCAAUGGUCAUAAUGCAtt	UGCAUUAUGACCAUUGAUGgt
131	hnRNPA2B1	s6715	Ambion Silencer Select	CCAUGAUCCUGUGGAUAAAtt	UUUAUCCACAGGAUCAUGGtc
132	hnRNPA3	s47926	Ambion Silencer Select	GCAAGAUUGAAACCAUAGAtt	UCUAUGGUUUCAAUCUUGCca
133	hnRNPA3	s47927	Ambion Silencer Select	GCUUUGAAACUACAGAUGAtt	UCAUCUGUAGUUUCAAAGCtc
134	hnRNPA3	s47928	Ambion Silencer Select	CAAGGUUGAUGGGCGUGUAtt	UACACGCCCAUCAACCUUGtg
135	hnRNPAB	s6716	Ambion Silencer Select	GGUUUAUCCUGUUCAAAGAtt	UCUUUGAACAGGAUAAACCca
136	hnRNPAB	s6717	Ambion Silencer Select	GGAUCCAAAGUUGAACAAAtt	UUUGUUCAACUUUGGAUCCat
137	hnRNPAB	s6718	Ambion Silencer Select	GAAGGACCCGGUGAAGAAAtt	UUUCUUCACCGGGUCCUUCtt
138	hnRNPC	s6719	Ambion Silencer Select	CAACGGGACUAUUAUGAUAtt	UAUCAUAAUAGUCCCGUUGaa
139	hnRNPC	s6720	Ambion Silencer Select	GAUGAAGAAUGAUAAGUCAtt	UGACUUAUCAUUCUUCAUCtc
140	hnRNPC	s6721	Ambion Silencer Select	ACACUCUUGUGGUCAAGAAtt	UUCUUGACCACAAGAGUGUtg
141	hnRNPCL1	s50975	Ambion Silencer Select	CACUCUUGUUGUCAAGAAAtt	UUUCUUGACAACAAGAGUGtt
142	hnRNPCL1	s50976	Ambion Silencer Select	GUUGAAAGGUGAUGACCUUtt	AAGGUCAUCACCUUUCAACtt
143	hnRNPCL2	s50974	Ambion Silencer Select	GCUUUCAACGGGAUUAUUAtt	UAAUAAUCCCGUUGAAAGCca
144	hnRNPD	s6722	Ambion Silencer Select	GAAUCGGAGAGUGUAGAUAtt	UAUCUACACUCUCCGAUUCtt
145	hnRNPD	s6723	Ambion Silencer Select	GGUUCUGCUUUAUUACCUUtt	AAGGUAAUAAAGCAGAACCca
146	hnRNPD	s6724	Ambion Silencer Select	GGAAGGUGAUUGAUCCUAAtt	UUAGGAUCAAUCACCUUCC
147	hnRNPDL	s19403	Ambion Silencer Select	CCCGGAUACUUCUGAAGAAtt	UUCUUCAGAAGUAUCCGGGct
148	hnRNPDL	s19404	Ambion Silencer Select	CAAAUUGAUAGAUCCCAAAtt	UUUGGGAUCUAUCAAUUUGcc
149	hnRNPDL	s19405	Ambion Silencer Select	GAACGAGUACAGCAAUAUAtt	UAUAUUGCUGUACUCGUUCat
150	hnRNPE1	s10094	Ambion Silencer Select	AGAUCAAAGAGAUCCGCGAtt	UCGCGGAUCUCUUUGAUCUta
151	hnRNPE1	s10095	Ambion Silencer Select	CACCAUUCCAAAUAACUUAtt	UAAGUUAUUUGGAAUGGUGag
152	hnRNPE1	s224175	Ambion Silencer Select	GGAAGUAGGAAGCAUCAUUtt	AAUGAUGCUUCCUACUUCCtt
153	hnRNPE2	s10097	Ambion Silencer Select	CCUUUGCUAUGAUCAUUGAtt	UCAAUGAUCAUAGCAAAGGct
154	hnRNPE2	s10098	Ambion Silencer Select	GCAGUAUCAUCGGAAAGAAtt	UUCUUUCCGAUGAUACUGCca
155	hnRNPE2	s10099	Ambion Silencer Select	CAGUCUCAUUUUCCCAUGAtt	UCAUGGGAAAAUGAGACUGtt
156	hnRNPE3 (PCBP3)	s28840	Ambion Silencer Select	GGGACCAAAAUCAAUGAAAtt	UUUCAUUGAUUUUGGUCCCtt
157	hnRNPE3 (PCBP3)	s28841	Ambion Silencer Select	CACCAUUCCCAAUGAUCUAtt	UAGAUCAUUGGGAAUGGUGag
158	hnRNPE3 (PCBP3)	s224291	Ambion Silencer Select	GAGAGGAUUGUGACCAUCAtt	UGAUGGUCACAAUCCUCUCtg
159	hnRNPE4 (PCBP4)	s32628	Ambion Silencer Select	AGAGCGAGCUGUUACGGUAtt	UACCGUAACAGCUCGCUCUgt
160	hnRNPE4 (PCBP4)	s32629	Ambion Silencer Select	UGAUUGCUUUCAAACUGGAtt	UCCAGUUUGAAAGCAAUCAtg
161	hnRNPE4 (PCBP4)	s32630	Ambion Silencer Select	GGGCGAGACUGUAAAGCGAtt	UCGCUUUACAGUCUCGCCCtt
162	hnRNPF	s6725	Ambion Silencer Select	GCAGCACAGAUAUAUAGAAtt	UUCUAUAUAUCUGUGCUGCat
163	hnRNPF	s6727	Ambion Silencer Select	CGACCGAGAACGACAUUUAtt	UAAAUGUCGUUCUCGGUCGct
164	hnRNPF	s230280	Ambion Silencer Select	GAAUGUAUGACCACAGAUATT	UAUCUGUGGUCAUACAUUCCG
165	hnRNPG (RBMX)	s56033	Ambion Silencer Select	GGCUUAAUACGGAAACAAAtt	UUUGUUUCCGUAUUAAGCCca
166	hnRNPG (RBMX)	s223747	Ambion Silencer Select	AGAUUAUACUUACCGUGAUtt	AUCACGGUAAGUAUAAUCUcg
167	hnRNPG (RBMX)	s26144	Ambion Silencer Select	CGUGAUGACUAUCCAUCAAtt	UUGAUGGAUAGUCAUCACGtg
168	hnRNPH1	s6728	Ambion Silencer Select	GAAGCAUACUGGUCCAAAUtt	AUUUGGACCAGUAUGCUUCaa
169	hnRNPH1	s6729	Ambion Silencer Select	GAGUAACUGGUGAAGCAGAtt	UCUGCUUCACCAGUUACUCtg
170	hnRNPH1	s6730	Ambion Silencer Select	GGAUUUGGGUCAGAUAGAUtt	AUCUAUCUGACCCAAAUCCat
171	hnRNPH2	s6732	Ambion Silencer Select	ACAGCUAUGUAGAACUUUUtt	AAAAGUUCUACAUAGCUGUga
172	hnRNPH2	s6733	Ambion Silencer Select	CAACCAGUCUAGUUAUGGAtt	UCCAUAACUAGACUGGUUGga
173	hnRNPH3	s226281	Ambion Silencer Select	CAUGCAACAUCGAUAUAUUtt	AAUAUAUCGAUGUUGCAUGtt
174	hnRNPH3	s6734	Ambion Silencer Select	CAUUGACGAUGGACUACCATT	UGGUAGUCCAUCGUCAAUGTT
175	hnRNPH3	s6736	Ambion Silencer Select	CGACCGGGACCAUAUGAUAtt	UAUCAUAUGGUCCCGGUCGct
176	hnRNPI (PTBP1)	s11434	Ambion Silencer Select	CAAAGCCUCUUUAUUCUUUtt	AAAGAAUAAAGAGGCUUUGgg
177	hnRNPI (PTBP1)	s11435	Ambion Silencer Select	CAGUUUACCUGUUUUUAAAtt	UUUAAAAACAGGUAAACUGat
178	hnRNPI (PTBP1)	s11436	Ambion Silencer Select	GCAUCACGCUCUCGAAGCAtt	UGCUUCGAGAGCGUGAUGCgg
179	hnRNPK	s6737	Ambion Silencer Select	CGAUGAAACCUAUGAUUAUtt	AUAAUCAUAGGUUUCAUCGta
180	hnRNPK	s6738	Ambion Silencer Select	GGUCAGCGGAUUAAACAAAtt	UUUGUUUAAUCCGCUGACCac
181	hnRNPK	s6739	Ambion Silencer Select	GAGCUUCGAUCAAAAUUGAtt	UCAAUUUUGAUCGAAGCUCcc
182	hnRNPL	s6740	Ambion Silencer Select	GGCUUGGAUCAAUCUAAGAtt	UCUUAGAUUGAUCCAAGCCat
183	hnRNPL	s6741	Ambion Silencer Select	GCACUCUGAAGAUCGAAUAtt	UAUUCGAUCUUCAGAGUGCaa
184	hnRNPL	s6742	Ambion Silencer Select	CGGAUGUUCUUUACACUAUtt	AUAGUGUAAAGAACAUCCGtg
185	hnRNPLL	s41040	Ambion Silencer Select	GUCUAAAUGUUAUUAGGAAtt	UUCCUAAUAACAUUUAGACga
186	hnRNPLL	s41041	Ambion Silencer Select	CCGUGUACAUUGCUGGUCAtt	UGACCAGCAAUGUACACGGgt
187	hnRNPLL	s41042	Ambion Silencer Select	CUGUUCUGCUUAUAUGGAAtt	UUCCAUAUAAGCAGAACAGgt
188	hnRNPM	s9259	Ambion Silencer Select	GAGAGAUCAUUGCAAAGCAtt	UGCUUUGCAAUGAUCUCUCct
189	hnRNPM	s9260	Ambion Silencer Select	GAAUAGGCACUGUUACUUUtt	AAAGUAACAGUGCCUAUUCca
190	hnRNPM	s9261	Ambion Silencer Select	GCUGCUAUUUGAUAGACCAtt	UGGUCUAUCAAAUAGCAGCtg
191	hnRNPP2 (FUS)	s5401	Ambion Silencer Select	AGCCCAUGAUUAAUUUGUAtt	UACAAAUUAAUCAUGGGCUgt
192	hnRNPP2 (FUS)	s5402	Ambion Silencer Select	GGUAAAGAAUUCUCCGGAAtt	UUCCGGAGAAUUCUUUACCat
193	hnRNPP2 (FUS)	s5403	Ambion Silencer Select	GGGUGAGAAUGUUACAAUUtt	AAUUGUAACAUUCUCACCCag
194	hnRNPQ (Syncrip)	s20562	Ambion Silencer Select	CUAUCGUGGUGGAUAUGAAtt	UUCAUAUCCACCACGAUAGtt
195	hnRNPQ (Syncrip)	s20563	Ambion Silencer Select	GAACGAGUGAAGAAGUUAAtt	UUAACUUCUUCACUCGUUCca
196	hnRNPQ (Syncrip)	s20564	Ambion Silencer Select	CAAUAGAGGUUAUGCGUUUtt	AAACGCAUAACCUCUAUUGag
197	hnRNPR	s19979	Ambion Silencer Select	GGUUAUACUCUGGAUGUAAtt	UUACAUCCAGAGUAUAACCag
198	hnRNPR	s19980	Ambion Silencer Select	GCACUGCGUAUGAAGAUUAtt	UAAUCUUCAUACGCAGUGCtt
199	hnRNPR	s19981	Ambion Silencer Select	GGAAAACUCGAAAGAGUAAtt	UUACUCUUUCGAGUUUUCCaa
200	hnRNPU	s6743	Ambion Silencer Select	CUACCUGGGUUACUAAACAtt	UGUUUAGUAACCCAGGUAGtt
201	hnRNPU	s6744	Ambion Silencer Select	GUAGAACUCUCGUAUGCUAtt	UAGCAUACGAGAGUUCUACtt
202	hnRNPU	s6745	Ambion Silencer Select	GAACUUCAGAGGACGAGGAtt	UCCUCGUCCUCUGAAGUUCtg
203	hnRNPUL1	s21883	Ambion Silencer Select	CUAAAAGACCGAACAAUAAtt	UUAUUGUUCGGUCUUUUAGgt
204	hnRNPUL1	s21884	Ambion Silencer Select	GAAACGCAACUAUAUCCUAtt	UAGGAUAUAGUUGCGUUUCtt
205	hnRNPUL1	s21885	Ambion Silencer Select	CAUGCGGUCUUAGAAAUGAtt	UCAUUUCUAAGACCGCAUGat
206	hnRNPUL2	s47964	Ambion Silencer Select	GGGCGAGAUUAUGAGUACAtt	UGUACUCAUAAUCUCGCCCgt
207	hnRNPUL2	s47966	Ambion Silencer Select	GAUUUUACGGGCGAGAUUATT	UAAUCUCGCCCGUAAAAUCGA
208	hnRNPUL2	s223522	Ambion Silencer Select	AGAGGAACUUUAUUCUUGAtt	UCAAGAAUAAAGUUCCUCUtt

Supplementary Table: ASO MOE reagents sequences used in the study

No	Sequence
1	CTCCCCACAGCCACT
2	CCCTCTCCCCACAGC
3	ACCCCTCTCCCCACA
4	CAACCCCTCTCCCCA
5	CCGACAACCCCTCTC
6	GCCCAGCCCGACAAC
7	AGCTCGGCCCAGCCC
8	CGAGGGTCAGCTCGG
9	GGAAACGAGGGTCAG
10	CTGTGGGGAAACGAG
11	GGCCCCTGTGGGGAA
12	GCCAGGGCCCCTGTG
13	GATCCATAGCCAGGG
14	AATTTGGATCCATAG
15	AGCCAGAAATTTGGA
16	ACTTGCAGCCAGAAA
17	TGCACTTGCAGCCAG
18	GACTCCTGCACTTGC
19	GTCACTGACTCCTGC
20	TGTACCGTCACTGAC
21	CCCTCCTGTACCGTC
22	ACCCTCCTGTACCGT
23	AACCCTCCTGTACCG
24	ACAAACCCTCCTGTA
25	ACGCACAAACCCTCC
26	ACGAGGACGCACAAA
27	TGCAGGGCACGAGGA
28	AGTGCAGGGCACGAG
29	TGGAAGAAAGTGCAG
30	TGGGATGGAAGAAAG
31	GGGTATGGGATGGAA
32	GTAGTAGGGTATGGG
33	CTTGTCGTAGTAGGG
34	GGAGTTCTTGTCGTA
35	ACTGGGGAGTTCTTG
36	CCATGAACTGGGGAG
37	CAGTAACCATGAACT
38	CGGAACCAGTAACCA
39	CTTCCCGGAACCAGT
40	GCTCCTTCCCGGAAC
41	GGCTCCTTCCCGGAA
42	TGGCTCCTTCCCGGA
43	TAATGGCTCCTTCCC
44	CTGGATATAATGGCT
45	GAGAGTCCCTGGATA
46	GGCCACTGGAGAGTC
47	TGTTTGTGGCCACTG
48	TCTAGCTTGTTTGTG
49	TTCTTGATCTAGCTT
50	TCCTGTACTTCTTGA
51	CTCCTCCTGTACTTC
52	AGTCTCCTCCTGTAC
53	GAGTCTCCTCCTGTA
54	TGAGTCTCCTCCTGT
55	TCTGCCCTGAGTCTC
56	AATCTGCCCTGAGTC
57	GGAATCTGCCCTGAG
58	AGGCGGAATCTGCCC
59	AGGAGGCGGAATCTG
60	ATCCCCAAGGAGGCG
61	TGTTCCTACTGGGAT
62	GCAGTTGTTCCTACT
63	CAGGGAGCAGTTGTT
64	CGATGCTCAGGGAGC
65	GCGTCTACGATGCTC
66	CTGGCGTCTACGATG
67	TCCTCCTGGCGTCTA
68	CCCTCCTCCTGGCGT
69	TCCCTCCTCCTGGCG
70	ATCCCTCCTCCTGGC
71	CCATTATCCCTCCTC
72	GTATGAACCATTATC
73	AAGAAGTATGAACCA
74	CCATCCGAAAGAAGT
75	CTCTCTCCATCCGAA
76	TCCTCTCTCCATCCG
77	ACTTCCTCTCTCCAT
78	GGTACTTCCTCTCTC
79	CTGTATTTGGTACTT
80	TTGTAACTGTATTTG
81	GGAGATTTGTAACTG
82	AGCTGGGGAGATTTG
83	ACAGAGAGCTGGGGA
84	CACATGCACAGAGAG
85	CTGTCACATGCACAG
NTC #1	TCGGTACCGACTGCA
NTC #2	GAATCGAAGTGCTAA
